# Surface Plasmon Resonance Imaging for Multidimensional Seed Vigor Phenotyping: From Single-Analyte Detection to Kinetic Fingerprinting

**DOI:** 10.3390/bios16100542

**Published:** 2026-09-28

**Authors:** Hao Zhang, Caisheng Xiao, Bo Wang, Huilong Fang, Zhengding Mei, Geng Zhou, Chen Chen, Zhanhong Zhang, Hongjun Xie, Yinghong Yu, Mingdong Zhu, Chenglong Guo, Youjun Zeng, Xiao Tang

**Affiliations:** 1 State Key Laboratory of Hybrid Rice, Longping Agricultural College, Hunan University, Changsha 410125, China; 2 Yuelushan Laboratory, Changsha 410128, China; 3 Institute of Cotton and Sericulture, Hunan Academy of Agricultural Sciences, Changsha 410125, China; 4 School of Basic Medical Sciences, Xiangnan University, Chenzhou 423000, China; 5 Hunan Vegetable Research Institute, Changsha 410125, China; 6 Hunan Agricultural Equipment Research Institute, Hunan Academy of Agricultural Sciences, Changsha 410125, China; 7 School of Physics and Optoelectronic Engineering, Guangdong University of Technology, Guangzhou 510006, China; 8 Guangdong Provincial Key Laboratory of Sensing Physics and System Integration Applications, Guangdong University of Technology, Guangzhou 510006, China

**Keywords:** surface plasmon resonance, SPR imaging, seed vigor, non-destructive testing, kinetic fingerprinting, biomarker panel, agricultural biosensing

## Abstract

Global food security faces escalating pressures from climate change, population growth, and resource limitations. Seed vigor, a complex physiological trait governing germination potential and stress resilience, remains critically underserved by conventional testing protocols that are destructive, time-intensive, and incompatible with industrial throughput. Surface plasmon resonance imaging (SPRi) offers a distinctive biosensing paradigm characterized by label-free detection, real-time kinetic analysis, and massively parallel multiplexing. Yet the agricultural SPR literature has been shaped by a single-analyte paradigm optimized for pesticide and pathogen detection, a framework that proves insufficient for the integrated interpretation of multiple biomarker panels that seed vigor demands. The biomarker panels considered here span macromolecular effectors that fall squarely within the direct detection range of SPRi and small-molecule osmoprotectants that are accessible through competitive, aptamer-based, or molecularly imprinted formats, a mixed-format multiplexing strategy for which experimental precedents on single sensor arrays exist. This review critically examines the operational boundaries of SPR/SPRi in agricultural matrices, surveys demonstrated performance across residue detection, soil monitoring, and breeding applications, and identifies why these achievements do not constitute a sufficient foundation for seed vigor assessment. We then analyze how SPRi technology must be strategically redesigned for non-destructive seed vigor evaluation, addressing biomarker selection criteria, non-invasive sampling hierarchies, surface functionalization for complex seed exudates, and kinetic phenotyping architectures for batch screening and stress-response fingerprinting. Performance enhancement through nanomaterial amplification, machine learning-based data interpretation, and microfluidic integration is evaluated alongside persistent gaps in matrix validation, biomarker standardization, and inter-laboratory reproducibility. The strategic significance of SPRi-enabled seed quality assurance for sustainable global agriculture is discussed in the context of these translational challenges.

## 1. Introduction

Global food security is under mounting pressure from climate change, population growth, and resource limitations [1]. Seed quality represents a fundamental determinant of crop productivity, yet conventional evaluation relies predominantly on standard germination tests requiring several days alongside destructive protocols such as tetrazolium staining, accelerated aging assays, and electrical conductivity measurements. These methods impose critical limitations on modern agricultural operations: extended analysis cycles preclude real-time quality monitoring during production, processing, and storage; consumed samples foreclose repeated testing or commercial use [2]; specialized personnel requirements restrict deployment in resource-limited settings [3]; and sequential processing impedes the large-scale screening essential for industrial seed operations [4,5].

The broader agricultural landscape encompasses chemical residue monitoring, foodborne pathogen detection, and soil and crop health assessment [6]. These domains share a common need for multiplexed analytical platforms that can operate reliably in complex matrices with minimal pretreatment [7]. Emerging biosensing technologies, including electrochemical sensors, fluorescence-based assays, Raman spectroscopy, and mass spectrometry, have demonstrated varying degrees of success [8]. Yet each approach carries inherent constraints: electrochemical methods frequently require redox-active labels [9]; fluorescence assays are susceptible to photobleaching and matrix interference [10]; Raman spectroscopy struggles with trace-level sensitivity [11]; and mass spectrometry demands infrastructure incompatible with field deployment [12].

Surface plasmon resonance (SPR) biosensing offers a fundamentally different approach. Distinguished by label-free operation, real-time kinetic analysis, and exceptional sensitivity to molecular binding events at sensor surfaces [13], SPR was first demonstrated for biomolecular detection in the early 1980s and has since revolutionized pharmaceutical research and clinical diagnostics [14]. Its high-throughput derivative, surface plasmon resonance imaging (SPRi), integrates charge-coupled device or complementary metal-oxide-semiconductor imaging to enable parallel monitoring of spatially resolved detection spots across two-dimensional sensor arrays [15]. Despite mature applications in biomedical and environmental monitoring, SPR/SPRi technologies remain significantly underutilized in agricultural contexts, particularly for seed quality assessment [16]. This gap is notable because the core capabilities of SPRi, including label-free detection, real-time kinetic recording, micrometer-scale spatial resolution, and parallel throughput, align precisely with the multidimensional requirements of seed vigor assessment, which demands non-destructive, multiplexed, and dynamic evaluation of a complex physiological trait rather than single-analyte endpoint detection.

The agricultural SPR literature has been shaped by a single-analyte paradigm: detect the pesticide, count the pathogen, quantify the hormone. These achievements, while substantial, have not addressed the integrated interpretation of multiple biomarker panels that seed vigor demands. This review therefore examines how SPR/SPRi technology must be strategically adapted, not merely repurposed, for the specific physiological complexity of seed vigor.

The choice of SPRi for seed vigor assessment warrants explicit justification, as the technique has been developed primarily for protein and pathogen detection rather than for plant physiological phenotyping. The justification rests on three grounds. First, the molecular effectors of seed vigor—heat shock proteins, LEA proteins, antioxidant enzymes, and nucleic acid integrity markers—are macromolecules well within the detection range of SPRi [17,18], while the small-molecule osmoprotectants in the panel are accessible through competitive, aptamer-based, or molecularly imprinted formats already validated for food-safety haptens [19,20,21]. The coexistence of such different detection formats on a single sensor array is documented practice rather than conjecture: sandwich and competitive immunoassays interrogating high- and low-molecular-weight toxins have been combined on a single patterned array [22]; multiple recognition elements have been co-immobilized on one SPR sensing surface for simultaneous multi-analyte analysis [23]; six-plex competitive detection of mycotoxins on a single imaging SPR chip has been validated in a cereal matrix [24]; and aptamer [25] and molecularly imprinted polymer [26] microarrays with SPRi readout have been demonstrated. Intact cells are not among the proposed vigor markers—vigor-relevant information is carried by molecules released into imbibition solutions, and membrane integrity is read out through macromolecular leakage rather than cell counts—but cellular targets are likewise within the demonstrated reach of SPR and SPRi, from direct whole-cell detection of seed-borne pathogens (Table 2) to single-bacterium counting and single-cell SPR microscopy [27]. Second, vigor is a temporally evolving physiological state, and among non-destructive analytical platforms, only SPRi provides real-time kinetic resolution of multiple specific biomarkers in parallel. Third, unlike spectroscopic imaging methods that infer vigor from statistical correlations, SPRi reports mechanistically interpretable binding events, enabling the transition from phenomenological classification to causal biomarker analysis. Table 3 benchmarks SPRi against established seed vigor assessment techniques across the operational dimensions relevant to industrial deployment.

Three review articles border this work without overlapping it. Reviews of SPR/SPRi instrumentation and biomedical or food-safety applications [12,28,29,30,31,32] do not treat seed science. Reviews of agricultural biosensing [5,11,33] survey sensor classes as catalog entries, without depth on kinetic analysis or seed-specific matrices. Reviews of seed vigor [2,4,17,18] synthesize physiological mechanisms and conventional or non-destructive testing, but label-free optical biosensing is absent from their methodological landscape. The present review occupies the intersection of these three literatures. Table 1 compares the scope of these three review streams with that of the present work.

**Table 1:** Scope comparison of this review with the three bordering review literatures.

Review Stream	Representative Reviews	Scope	Seed Vigor Coverage	Relation to This Review
SPR/SPRi instrumentation and applications	[12,28,29,30,31,32]	Optical principles, instrumentation, biomedical and food-safety applications	None	Physical and instrumental foundation synthesized in Section 2
Agricultural biosensing	[5,11,33]	Sensor classes for residue, pathogen, and crop monitoring	Catalog-level or absent	Application landscape critically surveyed in Section 3
Seed vigor and seed testing	[2,4,17,18]	Vigor physiology and conventional or non-destructive testing	Central, but label-free optical biosensing is absent	Physiological framework and benchmark methods used in Section 4 and Section 5
This review	-	SPR/SPRi mechanisms, agricultural applications, and seed vigor phenotyping	Central: mechanism-resolved vigor fingerprinting	Integrates the three streams; evidence status of each framework component mapped in Table 5

Unlike previous reviews focused on SPR instrumentation or food-safety single-analyte detection, this work critically examines why the dominant single-analyte paradigm is structurally insufficient for seed vigor—a polygenic, temporally evolving trait. The manuscript is organized as a critical evidence synthesis: Section 2, Section 3, Section 5 and Section 6 survey and evaluate 134 references across agricultural SPR/SPRi applications, performance enhancement strategies, and translational barriers; Section 4 and Section 7 present the framework that this synthesis yields, including a kinetic fingerprinting architecture and a systematic map of the evidence gaps for its realization. Each element of the proposed framework is anchored to the synthesized literature through the evidence-status matrix of Table 5. To our knowledge, this is the first review to position SPRi within the broader seed vigor phenotyping landscape.

Section 2 establishes the physical mechanisms and operational realities of SPR/SPRi. Section 3 critically surveys the demonstrated agricultural applications, evaluating the transferability of matrix-tolerant surface chemistry, physiological fluid compatibility, and limited multiplexing to the seed vigor context. Section 4 derives from this evidence base a technical framework for non-destructive seed vigor assessment, addressing biomarker selection, sampling strategies, surface functionalization, and kinetic phenotyping architectures; proposed elements are explicitly distinguished from demonstrated results throughout. Section 5 evaluates the demonstrated performance and documented failure modes of advanced enhancement strategies: nanomaterial amplification, machine learning, and microfluidic integration. Section 6 assesses the documented technical and institutional challenges of matrix complexity, biomarker validation, equipment accessibility, and data standardization. Finally, Section 7 synthesizes these threads into a forward-looking perspective on the future development and strategic significance of SPRi-enabled seed quality assurance.

Terminology. In this review, SPR denotes single- or few-channel resonance measurement, and SPRi denotes imaging-based array measurement. Following ISTA usage [2], seed vigor denotes the physiological potential for rapid, uniform emergence under diverse field conditions; it is distinct from viability, the binary capacity to germinate under optimal conditions, and from seed quality, the composite commercial attribute encompassing purity, health, and vigor. Sampling invasiveness follows the three-level scale defined in Section 4; “non-destructive” is used operationally to mean preserving germination capacity. Figure captions carry an evidence-status tag in square brackets, distinguishing experimentally demonstrated systems from conceptual architectures proposed in this work.

## 2. Fundamental Principles and Agricultural Performance of SPR/SPRi

### 2.1. Physical Mechanisms and Sensing Modalities

In the Kretschmann configuration, incident light undergoes total internal reflection at a prism–metal interface, generating an evanescent field that probes the adjacent dielectric to approximately 500 nm [28,29,34]. Molecular binding at the surface alters the local refractive index, producing a detectable shift in resonance angle or wavelength [30].

The real-time output of this transduction is a sensorgram that records the temporal evolution of the resonance signal as surface binding proceeds. Figure 1a illustrates this capability across 25 parallel sensing spots during anti-BSA antibody functionalization: each colored trace represents an independent channel, and the synchronized angle shifts confirm uniform surface coverage and binding consistency across the array [35]. Extending this concept, Figure 1b shows how modern SPR systems integrate multiple readout parameters, including dip position, spectral centroid, and AI-assisted model output, to deconvolute binding kinetics from instrumental noise, enabling reliable quantification even when biomolecular concentrations differ by orders of magnitude [36].

These readout capabilities are supported by diverse instrumental implementations. Figure 1c presents a compact prism-coupled system that integrates LED illumination, Kretschmann geometry, and microfluidic sample delivery in a miniaturized footprint [37]. For applications demanding richer optical information, Figure 1d depicts a polarization-modulated SPR imaging (Sp-SPRI) platform: the polarization state of the reflected beam is modulated and analyzed via Stokes vector extraction, yielding phase, amplitude ratio, and ellipticity maps in addition to conventional intensity changes [38]. Finally, Figure 1e demonstrates the high-throughput multiplexing potential of SPRi: four parallel microfluidic channels deliver distinct protein concentrations to spatially separated regions of the sensor surface, while the imaging system simultaneously records the resonance wavelength shift across the entire field of view, transforming single-channel kinetic measurement into spatially resolved, parallel phenotyping [39].

### 2.2. Capabilities of SPR/SPRi for Agricultural Analysis

SPR/SPRi offers four capabilities that are particularly relevant to agricultural quality assessment. First, label-free detection eliminates the need for fluorescent or enzymatic tags, preserving native analyte conformation and avoiding photobleaching and quenching [40,41,42]. Second, real-time kinetic recording yields association rate constants ($k_{a}$), dissociation rate constants ($k_{d}$), and equilibrium dissociation constants ($k_{D}$) without interrupting the measurement, providing mechanistic insight into molecular recognition [43]. Third, the non-destructive evanescent probing permits repeated interrogation of identical specimens, enabling longitudinal monitoring of physiological changes over time. Fourth, spatially resolved multiplexing allows simultaneous quantification of diverse targets within a single analytical run, transforming the sensor from a single-analyte detector into a multidimensional phenotyping platform [44,45,46,47]. It is the convergence of these four capabilities—rather than any single performance metric—that distinguishes SPRi from competing seed testing technologies, a comparison developed quantitatively in Section 4.1 (Table 3).

### 2.3. Demonstrated Performance in Agricultural Matrices

These capabilities have been translated into measurable analytical performance across several agricultural domains [31]. For pesticide and mycotoxin detection, SPR immunosensors have achieved picogram-per-milliliter sensitivity in buffer for organophosphorus compounds and sub-nanogram-per-milliliter for aflatoxin B1. Multi-channel configurations have enabled simultaneous detection of several pesticide classes or pathogen species, including *Listeria*, *Salmonella*, and seed-borne *Acidovorax avenae* [32,33,48]. In soil and crop monitoring, ionophore- and aptamer-modified sensors have quantified nitrate, phosphate, and heavy metals in soil extracts, and direct quantification of abscisic acid in tomato xylem sap has been validated against UPLC-MS/MS. In breeding applications, SPR-based nucleic acid detection has distinguished virus-infected from healthy plants within 30 min, and SPRi has screened mutant libraries for stress-resistance traits. Recent advances have further pushed sensitivity toward single-cell resolution, with single-bacterium counting demonstrated at 2 × 10^2^ CFU/mL [49].

### 2.4. Persistent Gaps Between Laboratory Performance and Agricultural Reality

Despite these achievements, significant gaps remain between demonstrated capability and deployable agricultural practice [50]. The picomolar detection limits cited above are predominantly obtained in simplified buffers or spiked extracts; the quantitative consequences of this discrepancy for real agricultural matrices are examined in Section 3.1. Antibody-functionalized surfaces face rapid degradation from proteases, polyphenol oxidases, and pH extremes endemic to agricultural matrices, yet chip regenerability under continuous use is rarely quantified [42]. Multi-channel configurations described as high-throughput rarely exceed ten fluidic channels, as quantified in Section 3.1.

Furthermore, the thermal and mechanical stability required for precise resonance tracking is difficult to maintain outside the laboratory. Temperature coefficients of approximately 0.01°/°C for resonance angle demand sub-degree stability that field-side or warehouse environments rarely provide. Vibration and particulate contamination introduce baseline drift that compromises kinetic parameter extraction. The theoretical throughput of 100 to 10,000 parallel spots stands in marked contrast to the modest parallelism actually deployed in agricultural studies, and microfluidic architectures capable of uniform sample delivery across large sensor footprints remain unresolved [51].

Most critically, the single-analyte paradigm identified in the Introduction cannot accommodate seed vigor, whose evaluation demands the integrated interpretation of multiple biomarker panels within a non-destructive, high-throughput framework [27]. The following section examines how the capabilities reviewed above have been applied across agricultural domains, and why the analytical paradigm that has served single-analyte detection proves insufficient for this more complex challenge. The Table 2 is representative limits of detection (LOD) for SPR/SPRi across agricultural domains.

**Table 2:** Representative limits of detection (LOD) for SPR/SPRi across agricultural domains.

Application Domain	Target Analyte	Matrix	LOD	Detection Strategy	Ref.
Pesticide residue	Phosmet	Peaches, apples, cabbages, rapeseed	1.6 ng/L (0.0016 ng/mL)	Competitive inhibition; PH-BSA immobilized chip	[52]
Mycotoxin	Aflatoxin B1	Corn, wheat	0.59 ng/mL	Competitive inhibition; SAM-based chip	[53]
	Ochratoxin A	Corn, wheat	1.27 ng/mL	Competitive inhibition; SAM-based chip	
	Deoxynivalenol	Corn, wheat	3.26 ng/mL	Competitive inhibition; SAM-based chip	
	Zearalenone	Corn, wheat	7.07 ng/mL	Competitive inhibition; SAM-based chip	
	Ochratoxin A (enhanced)	HBS-EP buffer	0.042 ng/mL	Au nanoparticle (40 nm) amplification; PEG-OVA conjugate	[19]
	Ochratoxin A	Oat	0.33 ng/g	Au nanoparticle enhancement; aqueous methanol extraction	
	Ochratoxin A	Corn	0.5 ng/g	Au nanoparticle enhancement; aqueous methanol extraction	
	Ochratoxin A	Grape juice	0.058 ng/mL	PVP pretreatment; competitive inhibition	
	Ochratoxin A	Red wine	0.33 ng/mL	PVP pretreatment; competitive inhibition	
Foodborne pathogen	Listeria monocytogenes	Culture medium	2 × 10^2^ CFU/mL	RO-SPRI; single-bacterium event counting; polyclonal antibody	[54]
Seed-borne pathogen	*Acidovorax avenae* subsp. *citrulli* (whole cells)	Culture medium	10^6^ CFU/mL	Direct detection; mixed SAMs; monoclonal antibody	[55]
	*Acidovorax avenae* subsp. *citrulli* (sandwich)	Culture medium	5 × 10^5^ CFU/mL	Sandwich amplification; polyclonal antibody enhancement	
Plant stress marker	Abscisic acid (ABA)	Running buffer	1.36 ng/mL	Direct non-competitive SPR immunoassay; anti-ABA antibody	[56]
	Abscisic acid (ABA)	Tomato xylem sap	5–90 ng/mL (quantified range)	Direct SPR immunoassay; validated against UPLC-MS/MS	

## 3. SPR/SPRi in Agriculture: A Critical Survey of Established Applications

The capabilities outlined in Section 2 have been applied across three major agricultural domains: chemical residue and pathogen detection, soil and crop health monitoring, and breeding and genetic selection. This section evaluates what has been achieved in each domain, identifies the persistent gaps between laboratory demonstration and field deployment, and explains why the accumulated achievements, while substantial, do not yet constitute a sufficient foundation for seed vigor assessment.

### 3.1. Chemical Residue and Pathogen Detection

SPR immunosensors for pesticides and mycotoxins have achieved impressive analytical sensitivity in controlled environments [57]. For organophosphorus and pyrethroid compounds, detection limits in the picogram-per-milliliter range have been reported [30], and competitive inhibition formats have enabled quantification of low-molecular-weight haptens including aflatoxin B1 and ochratoxin A [19]. Multi-channel sensor configurations have demonstrated simultaneous detection of multiple mycotoxin classes [53], and SPRi systems have distinguished foodborne pathogens such as *Listeria monocytogenes*, *Salmonella*, and *Escherichia coli* O157:H7 in food matrices, with reported equivalence to gold-standard plate culture methods [54].

For seed-borne pathogens, SPRi detection of *Acidovorax avenae* subsp. *citrulli* has been reported to surpass traditional PCR methods in speed and operational simplicity [55]. The detection architecture, illustrated in Figure 2, employs a mixed self-assembled monolayer surface functionalized with casein and monoclonal antibody to capture the pathogen within the evanescent field, with polyclonal antibody providing secondary signal enhancement [55]. This configuration exemplifies the antibody-based SPRi paradigm that has dominated agricultural pathogen detection.

However, these achievements rest on analytical conditions that diverge systematically from agricultural reality. The picogram-level limits of detection are predominantly obtained in simplified buffers or spiked extracts; when rigorous matrix-matched calibration is performed, sensitivity typically degrades by one to two orders of magnitude in real grain, vegetable, or soil samples [56]. Recovery rates and coefficients of variation in independently collected field samples are rarely disclosed. The antibody surfaces central to these assays face rapid degradation from proteases, polyphenol oxidases, and pH extremes endemic to agricultural extracts, yet the operational lifetime of a functionalized chip under continuous use is almost never quantified [58]. The multi-channel configurations described as high-throughput usually comprise only 2 to 10 fluidic channels, and critical engineering parameters including channel-to-channel carry-over, dead volume, and pressure-driven flow non-uniformity are seldom characterized. Furthermore, performance claims against PCR or plate culture often rest on mismatched sample preparation protocols, rendering direct comparison misleading [30].

These limitations reveal an analytical habit that runs deeper than any specific technical flaw: the agricultural SPR literature has optimized for detecting a single, known target in a complex matrix, and has treated matrix interference as a noise problem to be suppressed rather than as an information channel to be exploited. Beyond these conceptual limitations, a quantitative inconsistency demands scrutiny. Critical comparison reveals that reported LODs for identical targets (e.g., ochratoxin A) vary by one order of magnitude depending on surface chemistry, antibody clonality, and extraction protocols, yet head-to-head studies under standardized matrices are absent. Moreover, validation predominantly relies on spiked rather than naturally contaminated samples, and chip regenerability under continuous agricultural exposure is rarely quantified. These analytical fragilities are not confined to residue detection; they extend to any agricultural matrix where native physiological fluids replace simplified buffers.

### 3.2. Soil and Crop Health Monitoring

Soil nutrient monitoring and crop physiological assessment represent a conceptual advance over residue detection, because they treat the biological system itself as the analytical object rather than as an external contaminant. SPR sensors modified with ionophores or specific recognition elements have quantified nitrate, phosphate, and potassium in soil extracts, with reported detection limits of 0.1 μmol/L for nitrate and 0.05 μmol/L for phosphate [59]. Aptamer-based sensors have extended this capability to heavy metal contamination, achieving sub-nanogram-per-milliliter detection of Pb^2+^, Cd^2+^, and Hg^2+^ [60,61,62]. In crop health monitoring, direct quantification of abscisic acid in tomato xylem sap by SPR, validated against UPLC-MS/MS, demonstrates compatibility with plant physiological fluids and the feasibility of tracking stress-responsive metabolites in vivo [56,58].

The sigmoidal dose–response curve, spanning 1 to 1000 ng/mL with a high coefficient of determination (R^2^ = 0.9991), illustrates the quantitative dynamic range achievable for plant hormones under controlled conditions (Figure 3) [56].

Yet this progress introduces new tensions. Xylem sap collection is invasive, requiring stem excision or pressure extraction, which creates a logical contradiction with the non-destructive aspirations central to seed vigor assessment. If SPRi is to serve as a non-invasive phenotyping tool, the agricultural community must acknowledge that many validated crop-monitoring protocols are in fact destructive or semi-destructive [56]. Alternative sampling strategies such as leaf exudates or root secretions remain underdeveloped, and their compatibility with SPRi fluidic constraints has not been systematically evaluated.

In addition, high ionic strength in soil extracts alters the local refractive index in ways that are optically indistinguishable from specific binding, and few studies employ independent ionic-strength referencing to separate these contributions. The small-molecule detection strategies developed for plant hormones, including competitive inhibition formats and aptamer recognition, will be necessary for seed vigor markers such as abscisic acid and trehalose, both of which fall below the direct mass-detection limit of conventional SPRi [63].

Thus, while crop monitoring has moved closer to the physiological complexity of seed vigor, it has not yet resolved the sampling invasiveness or the small-molecule sensitivity gaps that seed matrices will present. If the transition from crop monitoring to seed quality assessment demands non-invasive operation, breeding applications introduce a different scaling challenge: the gap between laboratory spot counts and industrial throughput.

### 3.3. Breeding and Genetic Selection

In crop improvement, SPR-based nucleic acid detection and protein–protein interaction analysis have accelerated molecular marker-assisted selection. Virus-infected plants have been distinguished from healthy specimens within 30 min with reported accuracy exceeding 98% [64,65]. SPRi has been used to screen mutant libraries for stress-resistance traits, improving screening efficiency over conventional methods. The instrumental realization of this screening approach is exemplified by the Phytochip platform, which integrates a 17-spot gold sensor array with automated fluidic delivery and real-time imaging acquisition within a compact benchtop system (Figure 4) [64]. This architecture demonstrates the feasibility of array-based SPR detection for plant pathogen screening, yet the 17-spot configuration remains far below the throughput required for industrial-scale seed vigor assessment. These studies establish the technical feasibility of using SPR/SPRi as a screening platform in plant science, and they introduce the critical element of limited multiplexing [58].

Nevertheless, the multiplexing demonstrated in breeding studies, usually 2 to 5 markers, is modest compared with the multidimensional physiological complexity of seed vigor, which involves membrane integrity, oxidative metabolism, osmotic regulation, and genetic fidelity simultaneously. The reported accuracy figures are typically derived from small, laboratory-inoculated cohorts with artificial viral titers, and independent validation under field conditions with natural infection pressure remains sparse [58]. More importantly, the screening logic of breeding applications remains fundamentally diagnostic: it asks whether a specific marker is present or absent, and whether its concentration exceeds a threshold. It does not ask how multiple markers covary over time to encode a dynamic physiological state.

Collectively, the limitations identified across residue detection, crop monitoring, and breeding applications indicate that the agricultural SPR literature has developed necessary but insufficient capabilities for seed vigor assessment. The following section examines how these fragmented achievements must be strategically integrated and transcended.

### 3.4. Toward Seed Vigor Phenotyping

The transition from breeding diagnostics to seed vigor phenotyping is not merely a scaling challenge (more spots, more markers). It is a reconceptualization of what the sensor measures: from endpoint presence/absence to dynamic physiological state. Static multiplexing—quantifying multiple analytes at a single time point—cannot capture the temporal covariation of membrane deterioration, oxidative stress, and osmotic regulation that defines vigor. Only real-time kinetic recording, which SPRi uniquely provides among non-destructive seed testing technologies, can encode this dynamic state.

The agricultural SPR literature has developed three distinct capabilities across the domains reviewed above. From residue and pathogen detection, it has derived matrix-tolerant surface chemistry and antifouling strategies, including zwitterionic polymer brushes and on-chip reference subtraction. From soil and crop monitoring, it has established compatibility with physiological fluids and small-molecule detection formats. From breeding applications, it has demonstrated limited multiplexing and the feasibility of rapid screening.

Indeed, recent optical imaging studies have moved toward multidimensional phenotyping of seed vigor. Galletti et al. integrated multispectral data with principal component analysis to achieve cluster-based discrimination of seed lots with differing vigor levels in tomato and carrot (Figure 5) [63]. Yet this approach relies on morphological and spectral features rather than on the dynamic molecular interactions of specific biochemical markers. A systematic benchmark of SPRi against multispectral imaging and other non-destructive vigor assessment methods is provided in Section 4.1 (Table 3). SPRi, by combining multiplexed biomarker quantification with real-time kinetic recording, offers a pathway to translate such multidimensional phenotyping from statistical correlation to mechanistic resolution.

However, these capabilities have evolved in isolation, each optimized for a single-analyte, endpoint-detection, laboratory-validated paradigm. Seed vigor assessment demands their integration and their transcendence [63]. Unlike the analytical targets reviewed in Section 3.1, Section 3.2 and Section 3.3, seed vigor is a polygenic, temporally evolving physiological state [4] whose molecular basis is examined in Section 4.2; its evaluation requires the integrated interpretation of multiple biomarker panels within a non-destructive, high-throughput framework. The convergent requirements of multiplexed biomarker profiling, non-invasive operation, and dynamic longitudinal monitoring align precisely with the core capabilities of SPRi established in Section 2. But this intersection remains significantly underexplored, and the analytical tools developed for pesticide or pathogen detection cannot be transferred without fundamental adaptation.

Section 4 therefore examines how SPRi technology must be strategically redesigned, not merely repurposed, to address the specific physiological complexity of seed vigor. We analyze biomarker selection criteria grounded in SPRi transduction modalities, confront the sampling and surface-chemistry challenges unique to seed matrices, and translate SPRi’s imaging and kinetic capabilities into practical assay architectures for batch screening, aging trajectory tracking, and stress-response evaluation.

## 4. SPRi Technology for Non-Destructive Seed Vigor Assessment

Terminology of sampling invasiveness. Throughout this review, sampling strategies are classified on a three-level scale. Non-contact methods, such as headspace volatile capture, involve no physical interaction with the seed. Minimally invasive methods, principally imbibition sampling, preserve germination capacity as defined by standard germination testing but perturb metabolism: imbibition itself activates respiratory and repair pathways and can reshape the very marker profile under evaluation. Destructive methods, such as tissue homogenization, provide direct access to intracellular markers but foreclose longitudinal monitoring and commercial use. We use “non-destructive” in the operational sense of preserving germination capacity, with the metabolic-activation caveat stated wherever imbibition sampling is invoked.

The preceding sections have established that SPR/SPRi possesses the technical capabilities for complex agricultural analysis, and that the accumulated achievements in residue detection, crop monitoring, and breeding screening do not constitute a sufficient foundation for seed vigor assessment. This section translates the conceptual alignment between SPRi and seed vigor into a concrete technical framework, examining biomarker selection, sampling strategies, surface functionalization, and assay architectures while critically evaluating the gaps that separate theoretical promise from experimental demonstration.

### 4.1. Positioning SPRi Among Non-Destructive Seed Vigor Assessment Methods

Any candid assessment of SPRi for seed vigor must begin by acknowledging what established non-destructive methods already deliver. Near-infrared spectroscopy and multispectral or hyperspectral imaging provide rapid, genuinely non-contact classification of seed lots and have been validated across numerous crop species [66,67]; machine vision and X-ray imaging resolve morphological defects at industrial throughput [4,68]; chlorophyll fluorescence imaging reports maturity- and deterioration-linked physiological status within minutes [63,69,70]. Table 3 benchmarks these methods against SPRi across the operational dimensions that determine industrial utility.

The comparison clarifies both the niche and the cost of SPRi. Spectroscopic and imaging methods classify vigor through statistical correlations between spectral or morphological features and germination outcomes; they do not identify the molecular species whose abundance or dynamics underlie those correlations. SPRi occupies the complementary niche: it quantifies specific vigor-related biomarkers and, uniquely, records their release and binding kinetics in real time, converting vigor assessment from correlation-based classification to mechanism-resolved phenotyping. This capability is purchased at a price that optical methods do not pay: a sampling step (imbibition or headspace collection), surface functionalization consumables, and greater instrumental complexity. The realistic deployment scenario is therefore stratified—optical methods for first-pass high-throughput sorting, SPRi for mechanism-level grading, breeding selection, and storage monitoring where molecular information justifies the added cost.

**Table 3:** Benchmark comparison of SPRi with established seed vigor assessment techniques across the operational dimensions relevant to industrial deployment.

Method	Molecular Specificity	Kinetic Resolution	Multiplexing	Analysis Time	Throughput	Sample Preparation	Destructive	Field Applicable	Cost per Test
Standard germination test (ISTA)	None (endpoint phenotype)	No	No	7–14 d	Low	None	Consumes sample	No	Low
Tetrazolium staining	Indirect (enzyme activity)	No	No	1–3 h	Low	Staining, dissection	Yes	No	Low
Electrical conductivity	None (aggregate leakage)	Limited	No	24 h	Medium	Imbibition	Semi-destructive	Yes	Low
NIR spectroscopy [66,67]	Low (spectral overtones)	No	Implicit	<1 min	Very high	None	No	Yes	Low
Multispectral/hyperspectral imaging [63]	Low-moderate	No	Implicit	Minutes	High	None	No	Yes	Medium
Machine vision [4]	Morphological only	No	No	Seconds	Very high	None	No	Yes	Low
X-ray imaging [68]	Morphological only	No	No	Minutes	Medium	None	No	No	Medium
Chlorophyll fluorescence imaging [69,70]	Single pathway	Yes (single parameter)	No	Minutes	Medium	None	No	Yes	Medium
ELISA	High (single analyte)	No	Limited	2–4 h	Medium	Extraction	Yes	No	Medium
SPRi (this work)	High (biomarker-specific)	Yes (real-time, per spot)	Yes (hundreds of spots)	Hours (post-imbibition)	High potential	Imbibition + on-chip conditioning	No (viability preserved)	Emerging	Medium

### 4.2. Theoretical Compatibility and the Experimental Vacuum

SPRi offers four capabilities that are theoretically well suited to seed vigor assessment: label-free detection, real-time kinetic recording, spatially resolved multiplexing, and non-destructive probing [71]. These capabilities have been extensively demonstrated in the agricultural domains reviewed in Section 3, yet their deployment for seed vigor remains almost entirely theoretical. This section examines why the conceptual alignment between SPRi and seed vigor assessment is strong, and why the experimental translation has not yet occurred.

Seed vigor emerges from the coupled dynamics of membrane integrity, oxidative metabolism, osmotic regulation, and genetic fidelity [72,73]. Unlike the single-analyte targets reviewed in Section 3, it cannot be reduced to the concentration of a single molecule. Conventional seed quality evaluation relies on standard germination tests requiring several days, alongside destructive protocols such as tetrazolium staining and accelerated aging assays. These methods consume samples, preclude repeated testing, and provide only endpoint information that cannot capture the dynamic trajectory of physiological deterioration [74]. The industrial seed sector therefore requires an analytical platform that is simultaneously rapid, non-destructive, and information-rich.

SPRi meets these requirements through its convergent technical architecture [21]. Label-free operation eliminates analyte modification that would compromise physiological relevance. Real-time kinetic recording captures the dynamic molecular signatures of membrane deterioration, oxidative stress, and osmotic regulation as they unfold [75,76]. Micrometer-scale spatial resolution and parallel array detection enable simultaneous profiling of multiple biomarkers across hundreds of individual seeds [77]. The non-invasive probing mechanism preserves sample viability for longitudinal tracking and commercial utilization. Together, these characteristics offer a distinctive convergence of capabilities for translating the polygenic complexity of seed vigor into actionable quality data.

However, this positioning is largely theoretical. The agricultural SPRi literature contains no study that has actually deployed these capabilities for seed vigor assessment. The following sections therefore examine what would be required to do so, and where the current technical foundation proves adequate or inadequate.

### 4.3. Biomarker Panel Selection: From Physiological Mechanism to Detection Feasibility

Evaluating seed vigor through SPRi requires identifying biomarkers that are simultaneously physiologically informative and compatible with surface-confined refractive index sensing. Four marker classes have been proposed, each presenting distinct transduction challenges.

Proteinaceous markers, including heat shock proteins (HSPs), late embryogenesis abundant (LEA) proteins, and storage protein degradation products, typically exceed 15 kDa and are readily detectable through direct antibody-based binding on gold sensor surfaces [78,79]. The association kinetics, characterized by association rate constants ($k_{a}$) and equilibrium response levels ($R_{max}$), encode information beyond mere abundance [80]. Conformationally compromised HSPs may exhibit accelerated dissociation rates ($k_{d}$) due to impaired binding site integrity, a kinetic signature of protein dysfunction invisible to static endpoint assays. However, this kinetic discrimination has been demonstrated only in biomedical protein-folding studies; its correlation with seed germination potential remains experimentally unverified.

Reactive oxygen species metabolism markers present a more complex challenge. Superoxide dismutase (SOD), catalase (CAT), and peroxidase (POD) are proteinaceous enzymes detectable by antibody binding, yet SPRi measures enzyme abundance rather than catalytic activity. Establishing the abundance–activity correlation in seed exudate matrices, through parallel SPRi and standard enzymatic assays on identical samples, is therefore a prerequisite for interpreting these markers. The lipid peroxidation product malondialdehyde (MDA, ~72 Da) falls far below the direct detection limit of conventional SPRi, necessitating competitive inhibition formats or molecularly imprinted polymer recognition layers [29,81,82].

Osmoprotective metabolites, including trehalose (342 Da), proline (115 Da), and glycine betaine, share this small-molecule detection challenge [83]. Direct antibody-based detection is often impractical for these haptens; instead, aptamer-functionalized surfaces or molecularly imprinted polymer layers offer synthetic recognition alternatives with superior stability and selectivity. While these formats have been validated for food-safety haptens, their performance in polysaccharide-rich seed exudates must still be verified, particularly with respect to cavity accessibility and fouling resistance (Table 4).

Nucleic acid integrity markers exploit a fundamentally different transduction mechanism. Seed aging is accompanied by DNA fragmentation and stored mRNA degradation [84]. Figure 6 illustrates the nucleic acid detection assay based on SPRi hybridization. SPRi detection of short nucleic acid fragments (20 to 100 nucleotides) requires surface-immobilized capture probes, often locked nucleic acid analogs to enhance binding affinity and mismatch discrimination [77]. Although LNA probes have demonstrated mismatch discrimination for pathogen RNA, establishing their correlation with seed aging trajectories across defined accelerated-aging time courses is the critical next validation step [85].

D’Agata and Spoto originally developed this platform for nucleic acid genotyping, yet its underlying layout translates directly to seed vigor assessment. As shown in Figure 6, the sensor surface carries multiple discrete spots arranged in a spatially resolved array. Each spot is functionalized with a distinct capture probe, and the imaging system records binding signals from every spot simultaneously. The device therefore no longer behaves as a single-analyte detector but rather as a multidimensional profiling platform. This architecture naturally accommodates the four marker classes we propose here: protein integrity, ROS metabolism, osmoprotective metabolites, and nucleic acid stability. Immobilizing these probes together in spatially separated regions allows the system to capture parallel kinetic fingerprints of membrane status, oxidative stress, osmotic regulation, and genetic fidelity in one analytical run. That different marker classes require different detection formats does not preclude their integration on one chip: mixed sandwich and competitive formats on a single array [22], co-immobilized recognition elements on a single SPR surface [23], six-plex competitive imaging SPR in barley [24], and aptamer [25] and molecularly imprinted polymer [26] microarrays with SPRi readout have each been demonstrated experimentally. The engineering burden that this format heterogeneity introduces—compatible immobilization chemistries, spot-specific regeneration conditions, and cross-format cross-reactivity control—is acknowledged as a panel-integration challenge in Section 6.5. Table 4 maps each proposed marker class onto the detection format under which it has been experimentally demonstrated, together with the seed-matrix-specific property that remains to be verified.

**Table 4:** Proposed seed vigor biomarker panel: demonstrated detection formats, evidence base, species evidence, concentration status, and seed-matrix properties requiring verification.

Marker Class	Representative Markers	MW Range	Demonstrated SPRi Format	Evidence Base	Species Evidence	Concentration Status in Seed Exudates	To Verify in Seed Matrices
Protein integrity	HSPs, LEA proteins, storage protein fragments	>15 kDa	Direct antibody binding on gold	Stress protein content correlates with vigor [78]; proteome–vigor links [79,86]	Soybean [86]; Arabidopsis [79]	Not yet reported in seed exudates	Correlation with germination potential
ROS metabolism	SOD, CAT, POD	>20 kDa	Antibody binding (abundance)	Clinical immunoassays; seed oxidative physiology [18]	Multiple crop species (destructive assays) [18]	Enzyme release into exudate unquantified	Abundance–activity correlation
Lipid peroxidation	Malondialdehyde (MDA)	~72 Da	Competitive inhibition; MIP	Food-safety hapten assays [20,21,29,82]	Not demonstrated in seeds by SPRi	Exudate range unreported; tissue assays only	Cavity accessibility in polysaccharide-rich exudate
Osmoprotectants	Trehalose, proline, glycine betaine	115–342 Da	Aptamer/MIP recognition	Food-safety hapten assays [20,21]	Metabolite release documented across species [87,88]	Measurable in imbibition solution within 30 min [87,88]	Fouling resistance; batch reproducibility
Nucleic acid integrity	DNA fragments, stored mRNA	20–100 nt	LNA capture-probe hybridization	Pathogen RNA genotyping [77]; seed longevity mechanisms [84,85]	Arabidopsis (AtOGG1) [85]; dry-storage mRNA [84]	Not reported in exudates	Correlation with aging trajectories

Four constraints condition the deployment of these marker classes, and each defines a concrete validation objective. First, the correlation between SPRi-measured protein abundance and functional enzymatic activity, established in clinical immunoassays, must be re-established for seed matrices through parallel SPRi–enzymatic assay measurement. Second, panel size requires rational optimization: beyond three to five optimally selected markers, additional analytes may introduce noise without improving predictive accuracy, and marker selection should therefore be performed with orthogonal partial least squares or mutual information analysis on seed-specific datasets. Third, a panel predictive in cereals may not transfer directly to legumes because of divergent seed physiology, storage biochemistry, and stress response pathways [86]; cross-species equivalence mapping is required. Fourth, baseline marker expression varies with production environment, harvest moisture, and drying protocols, requiring normalization strategies anchored to reference seed lots.

### 4.4. Non-Destructive Sampling and Interfacial Engineering

Non-destructive sampling for SPRi seed vigor assessment operates on a continuum of invasiveness. Fully non-contact approaches such as headspace solid-phase microextraction capture volatile organic compounds emitted by seeds without physical interaction [81], yet SPRi detection of these volatiles requires gas–liquid conversion and suffers from sensitivity degradation relative to liquid-phase analysis [89]. Minimally invasive approaches such as imbibition solution analysis soak seeds in distilled water for 4 to 24 h, preserving germination capacity while generating sufficient sample volume; however, this procedure itself triggers metabolic activation, altering the marker profile under evaluation [90,91]. Destructive approaches such as tissue homogenization or sectioning provide direct access to intracellular markers but foreclose longitudinal monitoring and commercial use [92,93,94]. The following sections focus on the minimally invasive and non-contact strategies, as they alone satisfy the industrial requirement for viable seed preservation.

The surface chemistries required for seed vigor assessment must simultaneously suppress non-specific adsorption and provide selective recognition for target markers. Two complementary strategies have been developed for this purpose, as illustrated in Figure 7 [82,95].

The translation of biomarker detection into reliable SPRi operation requires confronting a matrix-specific challenge that distinguishes seed vigor assessment from the soil extracts and bacterial cultures discussed in Section 3. Seed exudates and imbibition solutions contain particulate debris, high concentrations of non-specific proteins and carbohydrates, and polyphenolic compounds that exhibit strong non-specific adsorption to gold surfaces. Without rigorous interfacial engineering, these components produce refractive index changes optically indistinguishable from specific marker binding [96].

Sampling protocols must therefore be designed with SPRi fluidic constraints in mind. Imbibition solution analysis, the soaking of seeds in distilled water at a standardized temperature (20–25 °C) for 4 to 24 h, remains the primary approach because it preserves seed viability while generating sufficient sample volume (100 to 500 μL per seed) [87,88] for SPRi flow-cell introduction. However, raw imbibition solutions cannot be injected directly into SPRi microfluidic channels. On-chip conditioning is required: microfiltration modules (0.22 μm pore size) integrated upstream of the sensor region remove particulate matter, while centrifugal microfluidic cartridges can perform automated debris separation and controlled dilution within a monolithic chip, reducing matrix viscosity to levels compatible with the laminar flow conditions required for stable SPRi baseline recording.

Headspace solid-phase microextraction captures volatile organic compounds (ethanol, acetaldehyde, ethylene) emitted by seeds as metabolic activity indicators [97]. However, SPRi transduction relies on refractive index changes at the liquid–solid interface, and gas-phase analytes produce signals one to two orders of magnitude weaker than equivalent liquid-phase concentrations after conversion. This sensitivity penalty, compounded by the low molecular weight of typical seed volatiles, means that headspace–SPRi coupling for vigor assessment remains conceptually attractive but analytically marginal. Integration with SPRi therefore demands not merely a gas–liquid interface conversion module where volatiles trapped on the fiber are thermally desorbed into a running buffer before passing over the sensor surface, but also signal amplification strategies that have not been demonstrated for agricultural volatile matrices [98].

Surface chemistry constitutes the critical defense against matrix interference. Self-assembled monolayers based on oligo(ethylene glycol)-terminated alkanethiols provide foundational antifouling protection by creating a sterically repulsive hydrated layer. Zwitterionic polymer brushes such as poly(carboxybetaine) and poly(2-methylacryloyloxyethyl phosphorylcholine) demonstrate superior resistance to polyphenol adsorption compared to conventional polyethylene glycol layers, owing to stronger surface hydration and electrostatically neutral charge distribution [75,95,99].

For small-molecule markers, molecularly imprinted polymers offer dual functionality: synthetic recognition cavities provide selectivity in carbohydrate-rich backgrounds, while the polymer matrix itself contributes to surface passivation [82,100].

Nevertheless, these strategies have been validated primarily in simplified buffer systems or food matrices, not in the complex seed exudates that would be encountered in vigor assessment. Zwitterionic coatings have demonstrated antifouling performance in food and clinical matrices [101]; the specific property that remains to be quantified is their long-term stability under continuous polyphenol exposure, which can be measured directly through accelerated fouling assays with real seed exudates. Molecularly imprinted polymers, while promising for small-molecule detection, suffer from batch-to-batch variability in cavity distribution and binding affinity that could compromise the reproducibility required for industrial grading [102]. Moreover, the assumption that imbibition preserves seed viability is partially compromised by the fact that soaking itself triggers metabolic activation, altering the very marker profile that the assay seeks to measure [103]. Imbibition at 20–25 °C for 4–24 h preserves germination capacity while releasing vigor-related markers at detectable concentrations [87,90,91]; primary metabolite exudation is detectable within the first 30 min of imbibition [88]. The specific property that remains to be quantified is the degree to which soaking-induced metabolic activation reshapes the marker profile within this window; time-resolved profiling of marker release over the first 24 h, benchmarked against non-contact headspace sampling, would directly close this gap.

### 4.5. High-Throughput Screening and Dynamic Aging Monitoring

The industrial seed sector requires vigor grading of large batches to inform quality control, inventory management, and market release decisions. SPRi addresses this demand through spatially resolved biomarker panel integration that generates multidimensional kinetic fingerprints for each seed sample.

A single SPRi sensor chip, typically 1 cm^2^ in area, can accommodate 96 to 384 discrete detection spots through microcontact printing or inkjet spotting of distinct biorecognition elements [104]. For seed vigor assessment, the array layout should be designed for physiological panel integration rather than arbitrary parallelism. A representative configuration dedicates spatially resolved regions to (i) a reference lane bearing antifouling polyethylene glycol brushes without capture molecules, enabling real-time background subtraction; (ii) a protein integrity panel (anti-HSP, anti-LEA) capturing membrane and metabolic status; (iii) an oxidative stress panel (anti-SOD, anti-CAT, anti-MDA-MIP) quantifying ROS burden; and (iv) an osmotic protection panel (aptamer-trehalose, MIP-proline) assessing cellular protection capacity [105]. This layout transforms a single SPRi measurement into a multidimensional vigor phenotype [106].

The information content of SPRi extends far beyond endpoint concentration values. Each detection spot records a complete binding kinetic curve comprising the association phase, equilibrium plateau, and dissociation phase. Grasso and colleagues demonstrated this parallel kinetic recording using a Y-shaped PDMS microfluidic flow cell assembled on an SPRi gold sensor. As shown in Figure 8a, the microfluidic network divides the incoming sample stream and delivers it to multiple detection regions simultaneously, providing the fluidic foundation for high-throughput panel screening [104]. Figure 8b shows the corresponding real-time reflectivity changes recorded from both signal and in-line reference regions [104]. The interaction trace resolves the full binding trajectory from initial association through equilibrium to final desorption, while the reference trace remains flat and corrects for background drift. This experimental architecture directly realizes the panel-based screening concept described above, where distinct biomarker probes immobilized across spatially separated spots generate parallel kinetic fingerprints under uniform flow conditions.

These sensorgrams yield quantitative parameters, including the association rate constant ($k_{a}$), dissociation rate constant ($k_{d}$), equilibrium binding response ($R_{max}$), and initial binding slope (dR/dt at t → 0), that collectively constitute a kinetic fingerprint unique to the vigor status of the seed sample. Unlike static quantification methods such as ELISA, which provide only concentration estimates, SPRi kinetic parameters capture molecular interaction quality [107]. A high $k_{a}$ combined with an elevated $k_{d}$ for HSPs may indicate denatured protein conformations with impaired chaperone function, a signature of deteriorating vigor invisible to endpoint assays [108,109,110].

Extracting biological information from these sensorgrams requires explicit separation of biological kinetics from sensing-related signal contributions. Four controls are standard in SPR methodology and are prerequisites for vigor fingerprinting: flow-rate variation tests, in which association rates independent of flow rate indicate kinetic control while flow-dependent rates indicate mass-transport limitation [43]; in-line reference channels for subtraction of bulk refractive-index changes and non-specific adsorption [104,111]; transport-corrected or two-compartment kinetic models where mass-transport limitation cannot be eliminated; and standardized imbibition, filtration, and temperature control to stabilize matrix viscosity and baseline drift. Kinetic parameters should be reported only from spots passing these controls. Under this discipline, a kinetic fingerprint is a comparative construct—interpretable as a difference between seed lots measured under identical fluidic conditions—rather than an absolute physical constant.

Throughput must instead be assessed over the complete sample-to-answer chain rather than by spot count alone: imbibition sampling (4–24 h, the dominant step), on-chip conditioning and injection, kinetic recording (90–120 min per array, a theoretical estimate under stated assumptions [112]), chip regeneration, and data analysis. Because imbibition batches can be prepared in parallel and array cycles pipelined, a realistic sustained capacity is two to three array cycles per instrument per day, corresponding to roughly 200–300 seed samples daily at 96 samples per array—still a decisive advantage over the 7–14 day latency of standard germination tests. Furthermore, SPRi’s continuous kinetic recording capability enables longitudinal, non-destructive monitoring of marker dynamics as aging unfolds. When the same seed batch is interrogated repeatedly at defined intervals, SPRi establishes individual marker decay curves. For protein markers such as HSPs and LEA proteins, $R_{max}$ typically exhibits a monotonic decline as membrane damage progresses, while MDA shows an ascending trajectory as lipid peroxidation intensifies [113]. Time-series SPRi data, when coupled with machine learning frameworks, can forecast the time-to-threshold, the interval until a critical marker concentration indicating the transition from high-vigor to substandard status is reached.

Realizing these projections requires closing three defined evidence gaps. First, array scale-up: agricultural SPRi studies overwhelmingly report fewer than 10 detection regions, and demonstrating a 96-spot vigor array with uniform sample delivery is the immediate engineering milestone. Second, kinetic robustness: association and dissociation parameters are sensitive to temperature, flow rate, and chip surface batch variations, and their stability in seed matrices must be characterized before kinetic fingerprints can be interpreted physiologically. Third, physiological validation: the initial binding slope must be shown to correlate with germination potential more strongly than endpoint concentration, and longitudinal monitoring protocols must be verified to preserve seed viability across repeated imbibition cycles. Each of these tests is executable with existing instrumentation.

### 4.6. Stress-Response Kinetic Fingerprinting

To interpret stress-response kinetic fingerprints measured by SPRi, it is necessary to understand the temporal hierarchy of plant stress signaling. The physiological cascade extends from rapid cellular events (seconds to minutes) through hormonal redistribution (hours) to whole-plant phenotypic changes (days) [105], as summarized in Figure 9.

During germination, seeds encounter environmental stresses including drought, temperature extremes, and salinity. Their capacity to withstand these conditions is largely determined by vigor level, and stress responses operate on timescales of minutes to hours: protein phosphorylation cascades initiate within minutes [113], heat shock protein synthesis peaks within 2 to 4 h [109], and osmolyte accumulation becomes significant within 6 to 12 h [91]. SPRi’s sub-minute temporal resolution and continuous flow capability are uniquely matched to capture these transient molecular events as they unfold at the sensor surface.

The experimental architecture integrates the seed sample within the fluidic pathway. The seed is positioned in a microfluidic chamber upstream of the SPRi sensor region [104]. Stress solutions, such as polyethylene glycol for osmotic drought simulation, NaCl for salinity stress, or temperature-controlled buffers for thermal stress, are perfused over the seed and subsequently directed to the sensor array. The SPRi system then monitors the real-time secretion kinetics of stress-responsive markers (proline, glycine betaine, small HSPs) into the perfusate. The critical analytical parameter is not the absolute equilibrium concentration of these markers, but their release dynamics: the initial slope of the binding curve (dR/dt in the first 60 to 120 s) reflects the speed of cellular stress response activation, a direct indicator of metabolic readiness.

Seeds possessing high vigor typically exhibit rapid, transient marker release characterized by steep initial binding slopes and rapid approach to equilibrium, reflecting robust membrane signaling and efficient transcriptional activation. Low-vigor seeds, by contrast, show delayed, attenuated, or prolonged release profiles with shallow initial slopes and sluggish equilibration, indicating compromised stress sensing and impaired protective metabolism [94]. SPRi kinetic fingerprinting can therefore distinguish these physiological states in real time, whereas endpoint assays would require destructive sampling at multiple arbitrary time points to approximate the same information.

This kinetic phenotyping capability extends to the evaluation of seed treatments. Priming, coating, or biostimulant applications aim to enhance stress tolerance, but their efficacy is conventionally assessed through post-germination survival assays requiring days. SPRi can evaluate treatment efficacy within hours by comparing pre- and post-treatment stress response kinetics: an effective treatment should restore the rapid transient release signature characteristic of high-vigor seeds.

Three aspects of this framework define the next round of experimental validation. First, the osmotic stress model: PEG-based simulation exerts physical membrane effects that differ from soil drying, and stress fingerprints should therefore be benchmarked against controlled soil-drying protocols. Second, two competing hypotheses predict opposite kinetic signatures—robust stress activation in high-vigor seeds versus accelerated leakage through compromised membranes in low-vigor seeds. Discriminating between them requires co-monitoring of macromolecular markers, released predominantly through membrane damage, and small osmolytes, released through active transport, a two-channel experimental design that SPRi arrays are uniquely positioned to execute. Kinetic shape analysis, distinguishing transient peaks from monotone accumulation, provides the second discriminating axis, and where ambiguity persists, conventional electrical-conductivity measurement of the imbibition solution serves as an orthogonal ground-truth benchmark for membrane integrity. Third, cross-species transferability: kinetic signatures established in model species must be validated in cereal and legume crops under harmonized stress protocols.

The framework developed in Section 4.2, Section 4.3, Section 4.4, Section 4.5 and Section 4.6 demonstrates that SPRi possesses the technical capabilities required for seed vigor assessment, yet nearly every component rests on theoretical inference rather than rigorous agricultural validation. Table 5 systematically maps the evidentiary status of each proposed technical component.

**Table 5:** Evidence status and validation gaps for SPRi-based seed vigor assessment.

Component	Approach	Evidence Status	Key Validation Gap
Protein markers	Anti-HSP/LEA kinetic capture	Biomedically feasible; seeds untested	Germination correlation across species
ROS/small molecules	Competitive/MIP for MDA, trehalose, proline	Food haptens validated	Seed matrix transfer; enzyme activity link
Nucleic acid integrity	LNA probes for fragmented DNA/RNA	Pathogen RNA validated	Aging correlation; species patterns
Sampling and monitoring	Imbibition/headspace; repeated interrogation	Conventional testing exists	Metabolic activation; cumulative stress
Array throughput	96–384-spot panels	17-spot demonstrated	Industrial flow uniformity; optical aberrations
Kinetic phenotyping	Multi-parameter sensorgram; stress-release kinetics	Theoretical	Validation against germination/field trials

The matrix reveals a systematic pattern: the biophysical building blocks have been individually validated in adjacent fields (biomedical diagnostics, food safety, plant physiology), but their integration within seed-specific matrices remains largely hypothetical. The most critical gaps are not instrumental; rather, they reside in the absence of cross-species, multi-season validation studies that correlate SPRi kinetic signatures with agronomic ground truth. Section 5 examines the advanced strategies that may begin to bridge this divide, while Section 6 assesses the institutional and technical barriers to closing it.

## 5. Advanced Strategies for Performance Enhancement

The validation gap identified in Section 4 is not irreducible. Three operational bottlenecks—trace small-molecule sensitivity, high-dimensional data interpretation, and complex sample handling—motivate the convergent enhancement strategies examined in this section. Section 5.1 addresses nanomaterial-mediated signal amplification, Section 5.2 examines machine learning for SPRi data interpretation, and Section 5.3 evaluates microfluidic integration toward automated and portable architectures. Each strategy is assessed first for its demonstrated capability, then for its specific limitations in the context of seed vigor assessment.

### 5.1. Nanomaterial-Enhanced Signal Amplification

The detection of biomarkers at the low concentrations typical of seed exudates often approaches the sensitivity limits of conventional SPRi. Nanomaterial-enhanced amplification leverages localized surface plasmon coupling, electromagnetic field enhancement, and mass-loading effects to magnify refractive index perturbations at the sensor interface [114].

Gold and silver nanoparticles are widely employed signal amplification agents. When target molecules bridge nanoparticle labels to the sensor surface, the resulting plasmonic coupling between the nanoparticle and the gold film produces a substantial resonance shift, effectively lowering the detection limit by one to two orders of magnitude. Graphene and its derivatives offer an alternative pathway through their exceptionally large surface area and facile functionalization, enabling dense immobilization of recognition elements that increase binding site availability and signal magnitude [115,116]. Metal-organic frameworks contribute high porosity and tunable surface chemistry, selectively enriching small-molecule markers such as trehalose or proline while simultaneously amplifying the optical response through refractive index modulation.

In seed vigor assessment, these strategies are particularly valuable because they counteract background interference from proteins, polysaccharides, and phenolic compounds in complex seed matrices. By amplifying the specific signal while surface chemistries suppress non-specific adsorption, nanomaterial enhancement improves the signal-to-noise ratio and extends the dynamic range to cover the full physiological spectrum from premium to substandard vigor.

Nanomaterial amplification has been demonstrated predominantly in simplified buffer systems, and its translation to seed matrices faces three testable risks: nanoparticle aggregation and protein corona formation in polyphenol-rich exudates, which can neutralize or invert signal enhancement; batch-to-batch variability in nanoparticle size and functionalization, which conflicts with industrial reproducibility requirements; and slow mass transport in metal-organic frameworks, which degrades the temporal resolution essential for kinetic fingerprinting. Each risk has a corresponding mitigation strategy—surface passivation of nanoparticles, statistical process control in synthesis, and thin-film MOF architectures—whose effectiveness in seed matrices defines the evaluation agenda for this strategy.

### 5.2. Machine Learning for SPRi Data Interpretation

SPRi generates inherently high-dimensional data: a single measurement produces a spatially resolved time series in which each detection spot records a complete binding kinetic curve. Manual interpretation becomes impractical when monitoring hundreds of array spots across multiple biomarkers and seed batches. Machine learning offers a systematic means to extract latent patterns and establish predictive relationships between SPRi signatures and seed vigor status.

Traditional machine learning approaches, including support vector machines and random forests, have demonstrated efficacy in classifying SPRi responses into discrete vigor categories based on engineered features such as equilibrium binding levels, association rate constants, and spatial intensity variance [117]. For more complex pattern recognition, convolutional neural networks process raw SPRi images as spatial maps, automatically learning hierarchical features that correlate with biomarker distribution and binding morphology without requiring explicit physical models [118]. Recurrent architectures, including long short-term memory networks, capture the kinetic evolution of binding events that encode information about analyte concentration and affinity [119].

A persistent challenge in agricultural biosensing is the scarcity of labeled training data, particularly when extending models across crop species or instrument platforms. Transfer learning mitigates this constraint by leveraging knowledge acquired from well-characterized model species to accelerate convergence in target domains with limited annotated data [120]. End-to-end deep learning pipelines further streamline the workflow by mapping raw SPRi sensorgrams or images directly to vigor grades or germination probability, bypassing intermediate quantification steps [121].

Yet the application of machine learning to SPRi seed vigor data remains largely theoretical. The overwhelming majority of machine learning–SPRi integration studies are retrospective, small-sample, single-laboratory investigations without independent validation sets; overfitting risk is consequently high. Published SPRi–ML studies are predominantly retrospective, with sample sizes in the tens to low hundreds and single-laboratory data collection; given that a 96-spot array generates on the order of 10^2^ kinetic features per sample, the feature-to-sample ratio makes overfitting the default risk rather than the exception. Credible deployment therefore requires nested cross-validation during model development, a fully external test set collected on different seed lots and production seasons, and pre-registered evaluation against germination ground truth. Models moving between platforms or species require domain adaptation or recalibration against local reference lots; the empirical evidence for this cross-platform reproducibility problem is examined in Section 6.4. Most critically, the “black box” nature of deep learning conflicts with the agricultural sector’s demand for interpretable decision-making: a seed grading system that cannot explain why a particular batch was classified as substandard will face regulatory and commercial resistance, regardless of its predictive accuracy.

### 5.3. Microfluidic Integration and System Automation

The translation of SPRi seed vigor assessment from laboratory bench to agricultural production environments depends critically on sample handling automation and system miniaturization. Microfluidic integration addresses both requirements by incorporating filtration, dilution, mixing, and reaction modules into monolithic chips that interface directly with the SPRi sensor [122].

The fabrication of these platforms has increasingly relied on additive manufacturing [116], which offers rapid prototyping and customizable geometries for complex fluidic networks. Representative implementations include 3D-printed sequential injection lab-on-valve (LOV) manifolds for automated sample handling and multiplexed detection [123], as illustrated in Figure 10.

In seed vigor applications, microfluidic cartridges perform automated sample preparation that is otherwise labor-intensive and variable [110]. On-chip filtration removes particulate debris from seed exudates, while controlled dilution channels adjust matrix complexity to levels compatible with the sensor surface chemistry. Integrated valving and pumping structures deliver sample aliquots to distinct array regions in a programmed sequence, enabling unattended multiplexed analysis [124]. These fluidic modules can be fabricated through soft lithography or three-dimensional printing, offering routes to low-cost disposable cartridges suitable for high-throughput industrial operation [123,125,126].

Beyond automation, microfluidic–SPRi integration provides a natural pathway toward portable and field-deployable systems. Replacing conventional laser sources with light-emitting diodes, compacting prism and coupling optics, and interfacing imaging sensors with smartphone platforms have yielded handheld SPR devices suitable for on-site sensing [127]. Pre-functionalized sensor chips and automated fluidic protocols eliminate the need for specialized technical personnel, while intuitive software interfaces guide operators through measurement and interpretation [128].

Translating these microfluidic platforms to seed vigor assessment requires resolving three engineering questions. Particulate debris and high-viscosity polysaccharides in seed exudates readily clog channels below 100 μm, and filter fouling under continuous operation must be characterized over realistic duty cycles. Additive manufacturing of cartridges introduces surface roughness and dimensional tolerances whose effects on fluidic sealing and kinetic reproducibility must be bounded by tolerance analysis. Finally, per-test consumable costs must be benchmarked against the economics of industrial seed grading, where thousands of samples are processed daily during harvest season; the portability–sensitivity trade-off of handheld designs is examined in Section 6.3.

### 5.4. The Integration Imperative and Its Remaining Barriers

The strategies examined in Section 5.1, Section 5.2 and Section 5.3 collectively extend the performance envelope of SPRi technology. Nanomaterial amplification addresses sensitivity limitations for detecting trace-level markers in complex matrices. Machine learning transforms high-dimensional kinetic data into actionable vigor classifications. Microfluidic integration automates sample handling and enables field deployment.

Yet these strategies are not additive in practice. Nanomaterial-enhanced assays generate more complex kinetic signatures that demand more sophisticated ML models, while ML models trained on laboratory data fail when confronted with the batch-to-batch variability of nanomaterial synthesis and microfluidic fabrication. Microfluidic systems designed for buffer-based assays require re-engineering to handle the viscosity and particulate load of seed exudates, and their integration with nanomaterial delivery modules introduces mixing uniformity challenges that have not been solved. The convergence of these three strategies into a single, robust seed vigor platform therefore requires not merely the sequential application of each enhancement, but their co-optimization under realistic agricultural constraints.

Moreover, none of these strategies addresses the fundamental validation gap identified in Section 4. They improve the technical performance of SPRi measurement, but they do not establish whether the measured biomarkers actually predict germination potential, field emergence, or stress resilience. Until rigorous, multi-site, multi-season validation studies correlate SPRi kinetic fingerprints with agronomic outcomes across diverse crop species and environmental conditions, the advanced strategies reviewed in this section remain solutions in search of a validated problem. Section 6 therefore examines the technical and institutional challenges that must be overcome to close this translational gap.

For seed vigor assessment specifically, the prioritization differs from general biosensing. Microfluidic standardization of seed exudate handling is the most urgent prerequisite, because nanomaterial enhancement and ML interpretation are both invalidated if sample delivery is non-uniform. Once fluidic reproducibility is established, ML-based kinetic fingerprinting should follow, as it requires stable baselines but does not depend on nanomaterial batch consistency. Nanomaterial amplification should be considered a tertiary enhancement, given the added complexity of protein corona formation in polyphenol-rich seed matrices.

## 6. Technical Challenges and Strategic Solutions

The performance enhancements examined in Section 5 substantially extend the analytical capabilities of SPRi for seed vigor assessment. Yet translating these laboratory advances into reliable agricultural practice requires navigating persistent constraints that transcend pure technical performance. This section examines four critical challenges: matrix complexity and non-specific adsorption (Section 6.1), biomarker validation and standardization across species (Section 6.2), equipment miniaturization and cost reduction (Section 6.3), and data standardization for inter-laboratory reproducibility (Section 6.4). Each challenge is evaluated first for the strategic solutions that have been proposed, then for the fundamental gaps that remain unresolved.

### 6.1. Matrix Complexity and Non-Specific Adsorption

The distinction between specific binding and non-specific adsorption is foundational to SPRi operation in complex matrices. Figure 11 illustrates this contrast using self-assembled monolayer (SAM) interfaces: upon carbonic anhydrase injection, the benzenesulfonamide-functionalized SAM yields a robust specific-binding signal, whereas the non-functionalized monolayer shows only a trivial bulk refractive-index response (Figure 11a); the ligand-presenting mixed SAM architecture on gold is shown in Figure 11b [129]. This comparison underscores why surface chemistry is the critical defense against matrix interference in seed vigor assessment.

Seed exudates and imbibition solutions present analytically hostile environments containing diverse proteins, carbohydrates, lipids, phenolic compounds, and ionic species at varying concentrations. Without adequate surface protection, these components adsorb non-specifically to gold sensor surfaces, producing refractive index changes that are optically indistinguishable from specific marker binding events. This interference is particularly insidious because non-specific adsorption often follows kinetics superficially similar to specific binding in initial phases, complicating real-time discrimination. Table 6 summarizes the principal interferent classes in seed-derived matrices, their interference mechanisms, and the corresponding mitigation strategies.

Addressing matrix interference demands a multifaceted strategy [131]. Zwitterionic polymers and polyethylene glycol brush layers create strongly hydrated surface films that sterically repel macromolecules while maintaining anchor points for biorecognition element immobilization [130]. Mixed self-assembled monolayers combining oligo(ethylene glycol) termination with reactive head groups offer a simpler but effective alternative [129]. Complementing surface passivation, sample pretreatment through microfiltration or size-exclusion chromatography can deplete high-molecular-weight interferents without compromising target marker integrity [132]. The imaging architecture of SPRi itself provides an intrinsic correction mechanism: reference spots lacking capture molecules, distributed across the sensor array, enable real-time background subtraction on a pixel-by-pixel basis [111]. Finally, the temporal signature of binding events offers a discriminatory dimension; specific binding typically exhibits characteristic association–dissociation kinetics that differ from the rapid but reversible accumulation characteristic of non-specific adsorption [133].

These strategies have proven effective in food safety and clinical diagnostics, where matrices such as serum or processed extracts have been successfully interrogated [101]. However, their translation to seed vigor assessment remains uncertain. Seed exudates contain polyphenol concentrations that exceed those of most food matrices by an order of magnitude, and the long-term stability of zwitterionic coatings under continuous polyphenol exposure has not been quantified. More critically, the reference spot correction assumes that non-specific adsorption is spatially uniform across the sensor array. In microfluidic channels with laminar flow, concentration gradients and particulate settling can produce strongly heterogeneous fouling patterns, with upstream regions experiencing substantially heavier adsorption than downstream regions. Under these conditions, global reference subtraction introduces systematic error rather than correcting it. The temporal discrimination strategy, while conceptually sound, requires binding kinetics to be measured over extended periods, yet industrial seed screening demands rapid turnaround that may not allow full kinetic characterization [133]. Matrix interference in seed vigor assessment has therefore been mitigated in principle; solving it in practice requires spatially resolved fouling characterization and rapid kinetic discrimination protocols validated in real seed exudates.

### 6.2. Marker Validation and Standardization

Robust biomarker validation requires demonstrating that SPRi signals can distinguish target analytes from matrix background at relevant concentrations. Figure 12 presents a representative example from food safety screening, where blank and spiked wheat samples are compared to establish cut-off values for multiple mycotoxins [134].

The biological complexity of seed vigor poses a validation challenge arguably more demanding than the analytical one. A biomarker panel predictive in cereal crops such as wheat, rice, or maize may not transfer directly to legumes or vegetables because of divergent seed physiology, storage biochemistry, and stress response pathways [134]. Establishing species-universal markers, or at least mapping species-specific marker equivalents, requires comprehensive multi-species validation studies that are currently sparse in the literature [133].

Beyond species boundaries, marker sensitivity must span the full vigor spectrum from premium to substandard, enabling both binary classification for commercial sorting and continuous grading for precision agriculture. This dynamic range is complicated by environmental modulation: marker expression levels vary with production environment, harvest moisture, drying protocols, and initial post-harvest quality. Robust predictive algorithms must therefore incorporate normalization strategies or environmental covariates rather than treating vigor as a fixed biochemical constant.

For commercial adoption, the validation framework must integrate SPRi marker quantification with comprehensive phenotyping protocols serving as ground truth. Standard germination tests, accelerated aging assays, field emergence trials, and seedling growth parameters each capture different facets of vigor [135]. Only by establishing rigorous predictive correlations across these reference methods can SPRi signatures acquire the credibility necessary for contractual quality assessment and regulatory acceptance.

Yet the current literature contains a fundamental epistemological trap. Most studies validate SPRi biomarkers by correlating them with germination rates, then infer that the biomarkers causally determine vigor [136]. This is a logical error: both the biomarker and germination rate may be parallel consequences of underlying aging damage, rather than the biomarker being a causal predictor of germination potential. Closing this causal gap requires intervention studies that manipulate biomarker levels independently and observe subsequent germination changes—a design exemplified by the demonstration that overexpression of the DNA repair enzyme AtOGG1 enhances seed longevity [85]—together with multi-site, multi-season trials powered to detect biomarker-by-environment interactions. Formal adaptation of International Seed Testing Association (ISTA) protocols to incorporate SPRi signatures would then provide the regulatory legitimacy that technical performance alone cannot confer.

### 6.3. Equipment Miniaturization and Cost Reduction

Despite the miniaturization advances described in Section 5, current SPRi systems remain predominantly laboratory instruments whose cost, size, and operational complexity present substantial barriers to deployment in seed production facilities, storage warehouses, or field stations. The optical subsystems, including prisms, coupling optics, high-resolution cameras, and temperature control modules, contribute disproportionately to both footprint and expense [128].

Miniaturization of SPRi platforms depends on integrating optical, fluidic, and sensing layers into compact, manufacturable architectures. Figure 13 illustrates a representative approach employing a three-layer stacked structure: a nanohole array plasmonic sensing layer, a microfluidic delivery layer, and a broadband OLED illumination layer [137].

Several engineering pathways are converging to address these barriers. Light-emitting diode illumination, compact molded prisms, and smartphone-connected imaging sensors have already enabled handheld SPR device prototypes [47]. For disposable components, roll-to-roll processing of polymer sensor films and three-dimensional printing of microfluidic cartridges offer manufacturing paradigms that could reduce per-test costs to levels compatible with routine seed quality control [125,137]. Automated fluid handling, pre-functionalized sensor chips shipped in sealed cartridges, and guided software interfaces further lower the operational threshold.

However, the cost structure of SPRi seed vigor assessment is more complex than the hardware bill of materials suggests. The functionalized sensor chip, with its antibody or aptamer recognition layer, and the microfluidic cartridge, with its integrated filtration and dilution modules, represent recurring consumables whose cost per test may exceed the depreciation of the optical instrument itself. In industrial seed operations, where thousands of samples must be processed daily during harvest season, consumable economics dominate total cost of ownership. The handheld prototypes demonstrated to date have achieved portability by simplifying optical and thermal subsystems, but this simplification typically degrades sensitivity and baseline stability in ways that are incompatible with the low-abundance markers and kinetic precision required for vigor fingerprinting. Furthermore, the batch-to-batch variability introduced by additive manufacturing (Section 5.3) offsets its capital-cost advantages through quality-control overhead until statistical process control suitable for industrial metrology is achieved [123].

### 6.4. Data Standardization and Inter-Laboratory Reproducibility

An emerging constraint that has received limited attention in the SPRi literature concerns data standardization and inter-platform reproducibility. Unlike established analytical techniques with unified readout standards, SPRi systems report resonance changes in heterogeneous units, including angular shifts, wavelength displacements, or normalized intensity variations, depending on the optical configuration. Imaging algorithms for spot identification, background correction, and kinetic extraction vary widely between commercial platforms and are rarely documented with the granularity required for independent replication [111].

This heterogeneity becomes acute when machine learning models are transferred across instruments. A classification algorithm trained on a prism-based angular-readout system may degrade substantially when deployed on a grating-coupled intensity-modulation platform, even when identical biorecognition chemistry is employed [138]. Rath and colleagues directly quantified this cross-platform variability by measuring identical aptamer–target interactions across five label-free biosensing systems. As shown in Figure 14a, normalized binding curves for the same aptamer diverge in shape and temporal resolution depending on the instrument platform, compromising the reproducibility of kinetic parameter extraction [138]. Figure 14b summarizes equilibrium dissociation constants measured by microscale thermophoresis (MST) for the six thrombin aptamers, and Figure 14c maps the association and dissociation rate constants of the same aptamer–target pair across SPR, BLI, iRIf, SCORE, and SPRi. Together they show that both affinity values and kinetic parameters for identical aptamer–target pairs span orders of magnitude across platforms, confirming that heterogeneous reporting units and imaging algorithms introduce systematic bias [138]. This empirical evidence underscores why machine learning models trained on one platform configuration cannot be reliably deployed on another without absolute refractive index calibration and standardized kinetic extraction protocols.

The current absence of publicly available benchmark datasets further impedes algorithmic comparison and cross-laboratory validation.

Systematic solutions must be pursued at the community level. Signal calibration protocols traceable to absolute refractive index units would enable direct comparison across hardware configurations. Open-source algorithm repositories and shared benchmark datasets, modeled after successful initiatives in genomic and imaging informatics, would accelerate methodological convergence. Most critically, formal calibration against ISTA standard germination protocols would anchor SPRi vigor indices to the regulatory frameworks governing global seed commerce, providing the metrological legitimacy necessary for commercial adoption [135].

Yet these standardization efforts face a chicken-and-egg dilemma. Benchmark datasets cannot be created without validated, cross-platform measurement protocols, but such protocols cannot be validated without reference datasets. The commercial SPRi instrument manufacturers have little incentive to disclose proprietary imaging algorithms or adopt common calibration standards, as their business models rely on platform lock-in [139]. The academic community, which might otherwise drive open standards, lacks the coordinated infrastructure and funding mechanisms that enabled analogous efforts in genomics. Consequently, data standardization for SPRi seed vigor assessment remains a recognized necessity without a clear pathway to implementation.

### 6.5. The Translational Gap as a Systemic Problem

A further integration challenge accompanies the format heterogeneity of the proposed biomarker panel. Because the panel spans macromolecular markers detected by direct binding and small-molecule osmoprotectants detected through competitive, aptamer-based, or molecularly imprinted formats (Section 4.3, Table 4), a single vigor chip must accommodate different immobilization chemistries, different regeneration conditions, and different signal amplitudes on spatially separated spots operated under one flow protocol. Mixed-format arrays of this kind have experimental precedents [22,23,24,25,26], but each published demonstration addressed a narrower analyte panel than the four-class architecture proposed here; the compatibility of, for example, MIP regeneration solvents with antibody-bearing spots on the same chip remains unverified. We therefore regard mixed-format panel integration as an engineering milestone in its own right—intermediate between the single-format multiplexing already demonstrated [23,24] and the full kinetic fingerprinting architecture of Section 4—and list it among the foundational-tier research priorities in Section 7.2.

The challenges examined in Section 6.1, Section 6.2, Section 6.3 and Section 6.4 are not independent obstacles but interconnected elements of a single translational gap. Matrix complexity (Section 6.1) determines which biomarkers can be reliably detected, but the biomarker validation framework (Section 6.2) determines whether those detections predict agronomic outcomes. Equipment miniaturization (Section 6.3) determines whether the assay can be deployed at scale, but data standardization (Section 6.4) determines whether results obtained at one location can be trusted at another. Overcoming these barriers demands coordinated progress across surface chemistry, plant physiology, optical engineering, and data science, a level of interdisciplinary integration that the current funding and publication structures do not readily support.

Section 7 synthesizes these threads into a forward-looking perspective on the future development and strategic significance of SPRi-enabled seed quality assurance, identifying priorities for research investment and institutional coordination.

## 7. Conclusions and Outlook

### 7.1. Summary of Core Capabilities

This review has traced the trajectory of SPR/SPRi technology from fundamental optical principles through broad agricultural applications to the specific frontier of non-destructive seed vigor assessment. The analysis establishes that SPRi possesses a distinctive convergence of capabilities that directly address the critical limitations of conventional seed vigor evaluation: label-free operation eliminates reagent preparation and analyte modification; real-time kinetic recording captures dynamic molecular signatures of membrane deterioration, oxidative stress, and osmotic regulation; micrometer-scale spatial resolution and parallel array detection enable simultaneous profiling of multiple biomarkers across hundreds of individual seeds; and the non-invasive probing mechanism preserves sample viability for longitudinal tracking and commercial utilization. As analyzed in Section 4, Section 5 and Section 6, these capabilities converge on a single function that conventional vigor testing lacks: real-time, multiplexed, mechanism-resolved phenotyping of viable seeds.

### 7.2. Future Directions

Realizing this potential requires coordinated advancement across four fronts of research and development, each addressing distinct translational gaps identified in Section 6.

Four priorities must be addressed in parallel. First, multi-omics marker discovery must bridge the divide between omics-scale discovery and SPRi-compatible detection. While transcriptomic, proteomic, and metabolomic analyses can identify thousands of differentially expressed features, SPRi can practically monitor only a handful of markers per chip. Establishing the prioritization criteria for reducing this complexity to a deployable panel and the normalization strategies required for environmental sensitivity across production zones are the first deliverables of this tier. A critical priority within this tier is resolving the two logical paradoxes identified in Section 4: the metabolic activation induced by imbibition sampling, which demands the development of minimal-activation protocols such as low-temperature short-duration soaking or volatile headspace capture; and the membrane-leakage ambiguity, which requires multi-parameter kinetic signatures rather than single-metric interpretation to distinguish compromised membrane integrity from robust stress response. This priority involves implementing the two-channel discrimination design of Section 4.6, benchmarking co-monitored macromolecular leakage markers and small osmolytes against electrical-conductivity measurements as orthogonal ground truth.

Second, surface chemistry innovation must advance from biocompatibility to agricultural survivability. Antifouling zwitterionic coatings, nanomaterial-enhanced substrates, and synthetic recognition elements such as aptamers and molecularly imprinted polymers have demonstrated promise in simplified buffers, and their long-term stability under continuous exposure to polyphenol-rich, enzymatically active seed matrices must be quantified through accelerated aging of functionalized chips. Batch-to-batch variability in affinity and selectivity further complicates industrial standardization.

Third, intelligent system integration demands the convergence of SPRi detection with microfluidic sample handling, machine learning-based interpretation, and Internet-of-Things connectivity. However, the data standardization required for cloud-based model sharing is blocked by the absence of common calibration protocols and the commercial incentives for platform lock-in. Machine learning models require training datasets that do not yet exist, and their black-box nature conflicts with regulatory demands for interpretable decision-making.

Fourth, equipment democratization through handheld devices and low-cost manufacturing faces a fundamental economic paradox. The optical and thermal simplifications that enable portable designs degrade the sensitivity and baseline stability required for multiplexed kinetic fingerprinting, while the disposable sensor chips and microfluidic cartridges that would make portable operation practical represent recurring consumables whose cost per test may exceed the depreciation of the instrument itself.

### 7.3. Strategic Significance

SPRi-enabled seed vigor assessment carries implications that extend well beyond quality control laboratories. Non-destructive, high-throughput phenotyping will accelerate breeding programs by enabling rapid selection of superior genotypes without consuming valuable seed stock. Real-time storage monitoring will reduce post-harvest losses and optimize inventory management through data-driven decisions on temperature, humidity, and turnover timing. Precision sowing decisions informed by vigor data will enhance resource use efficiency, improve crop uniformity, and reduce the risk of establishment failure under suboptimal field conditions.

The convergence of SPRi biosensing with digital agriculture infrastructure establishes the technical foundations for comprehensive seed quality assurance systems essential to global food security. Yet this transition will not occur through technical advancement alone. It demands the coordinated resolution of the validation, standardization, and cost barriers examined in Section 6, and the institutional will to move from isolated proof-of-concept studies to multi-site, multi-season, pre-registered validation trials. The future of SPRi-enabled seed vigor assessment is therefore not merely a question of what the technology can do, but of whether the agricultural and scientific communities can align their incentives to prove what it has done.

## Figures and Tables

**Figure 1:**
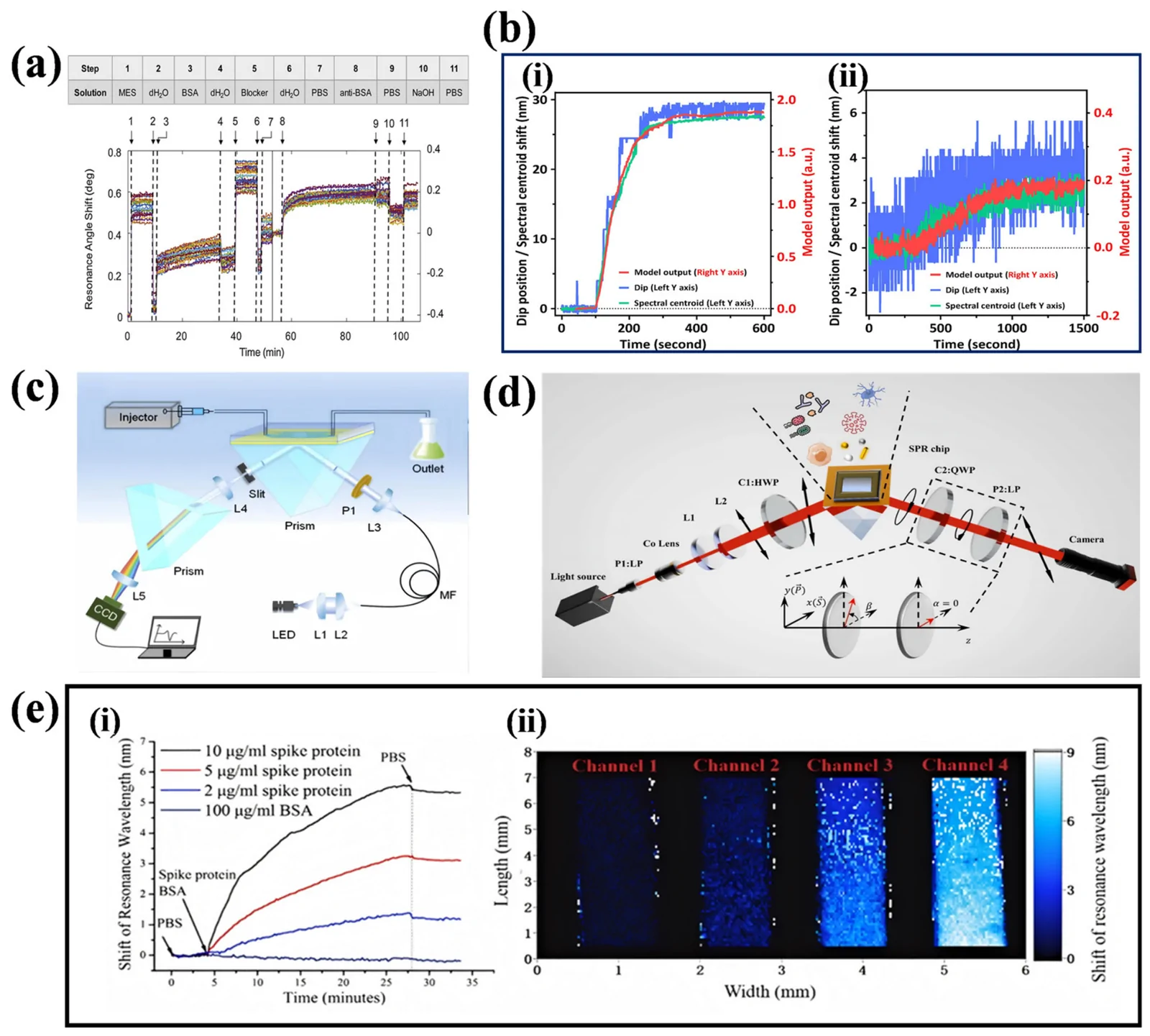
Representative SPR and SPRi instrumentation and readout modalities. (**a**) Real-time surface functionalization monitoring across 25 sensing spots, illustrating multi-channel kinetic consistency [Experimentally demonstrated system, adapted from Ref. [35]]. (**b**) SPR sensorgrams displaying dip position, spectral centroid, and AI model output for high- and low-concentration biomolecular binding [Experimentally demonstrated system, adapted from Ref. [36]]. (**c**) Compact SPR system with Kretschmann configuration, LED illumination, and microfluidic sample delivery [Experimentally demonstrated system, adapted from Ref. [37]]. (**d**) Schematic of a polarization-modulated SPR imaging platform based on Kretschmann geometry [Experimentally demonstrated system, adapted from Ref. [38]]. (**e**) Microfluidic-channel λSPR images and corresponding resonance wavelength shifts for multiplexed protein detection at varying concentrations [Experimentally demonstrated system, adapted from Ref. [39]].

**Figure 2:**
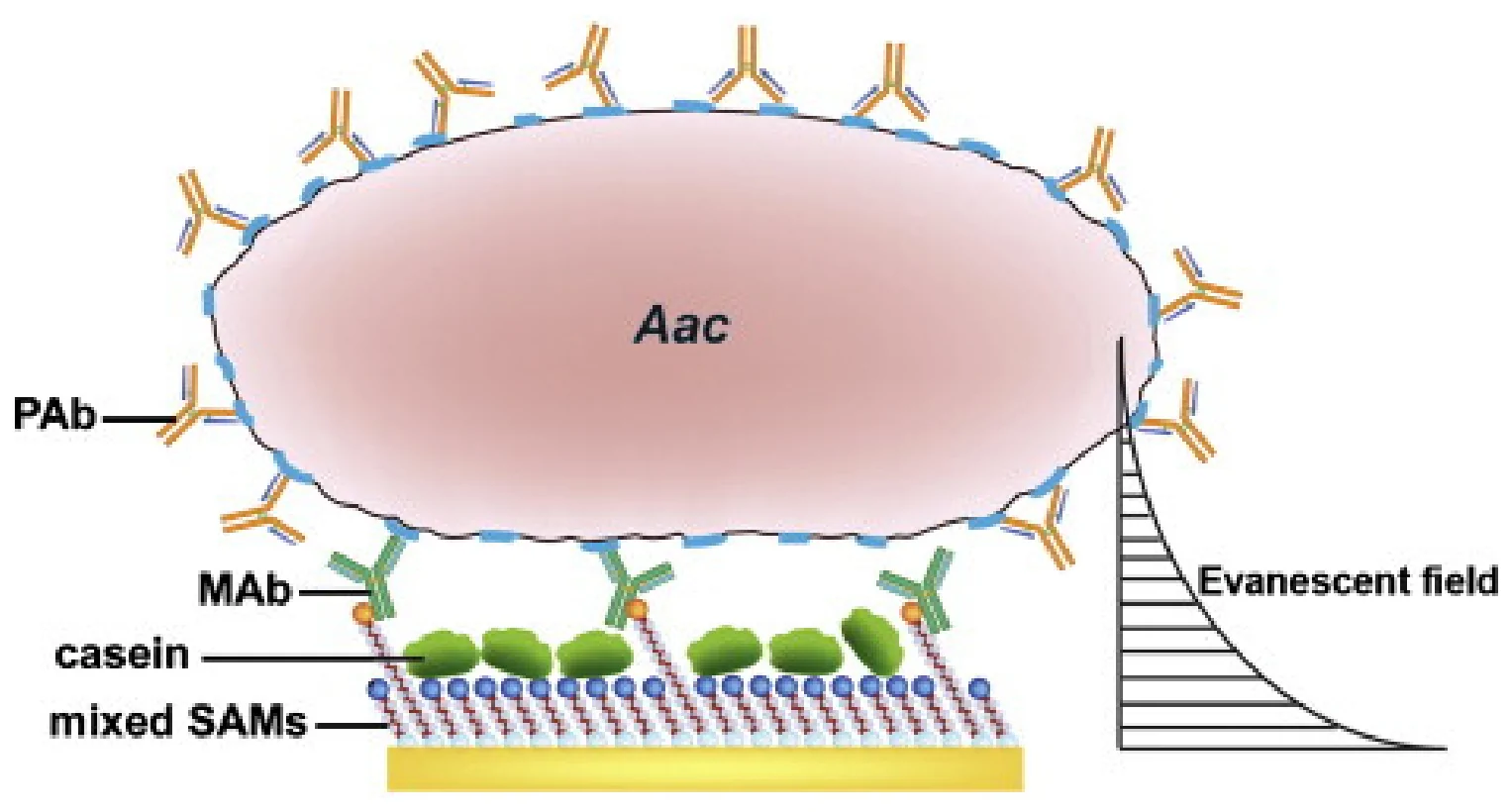
Schematic of SPRi-based detection of *Acidovorax avenae* subsp. *citrulli* (Aac) using a mixed self-assembled monolayer (SAM) surface. Casein and monoclonal antibody (MAb) are immobilized on the gold sensor surface to capture the pathogen, while polyclonal antibody (PAb) binds to the bacterial surface for enhanced detection, all within the evanescent field penetration depth [Experimentally demonstrated system, adapted from Ref. [55]].

**Figure 3:**
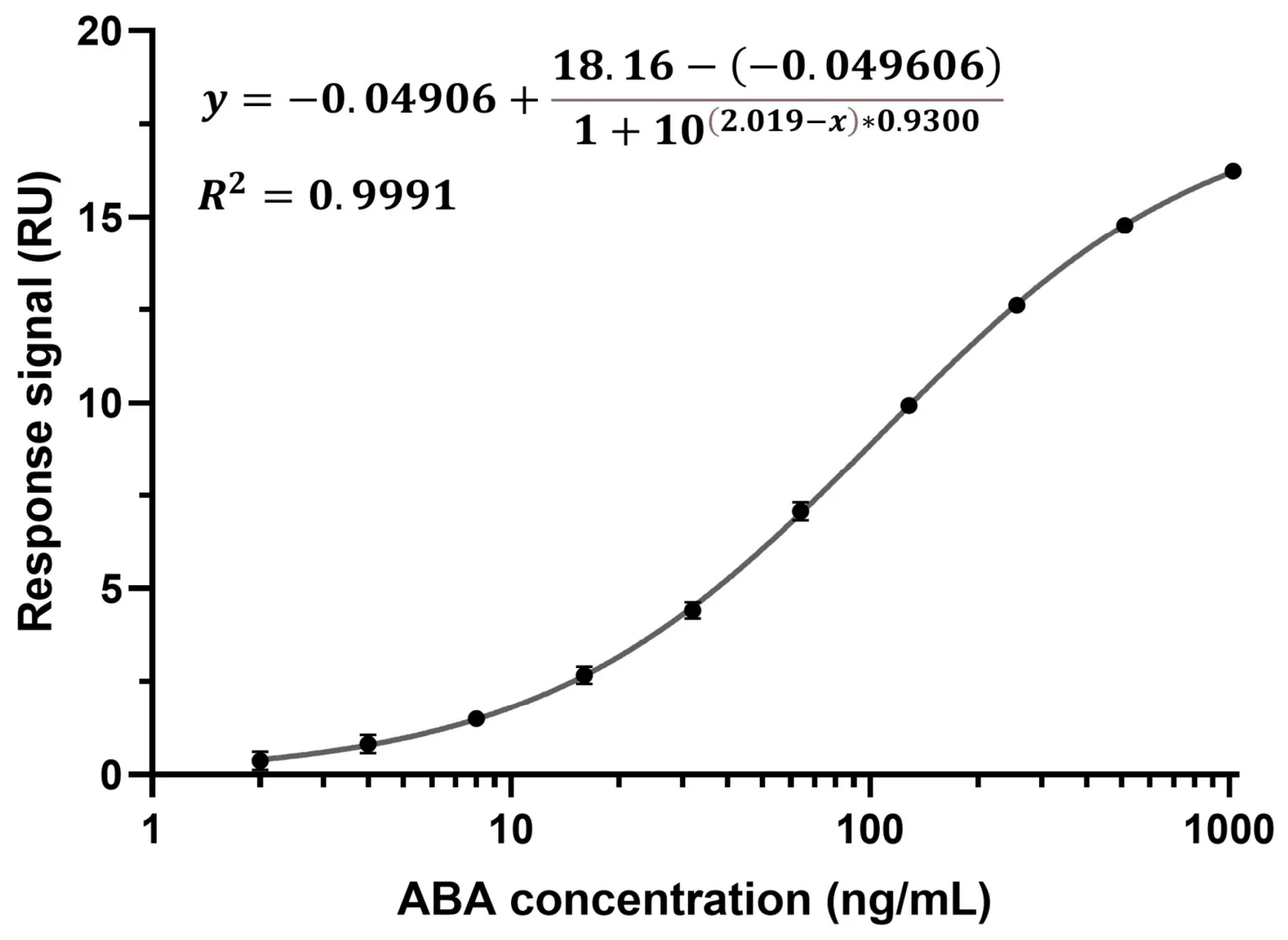
Standard dose–response curve for abscisic acid (ABA) quantification by SPR, showing response signal (RU) versus ABA concentration (ng/mL) on a logarithmic scale. The fitted sigmoidal curve spans the dynamic range from 1 to 1000 ng/mL with R^2^ = 0.9991 [Experimentally demonstrated system, adapted from Ref. [56]].

**Figure 4:**
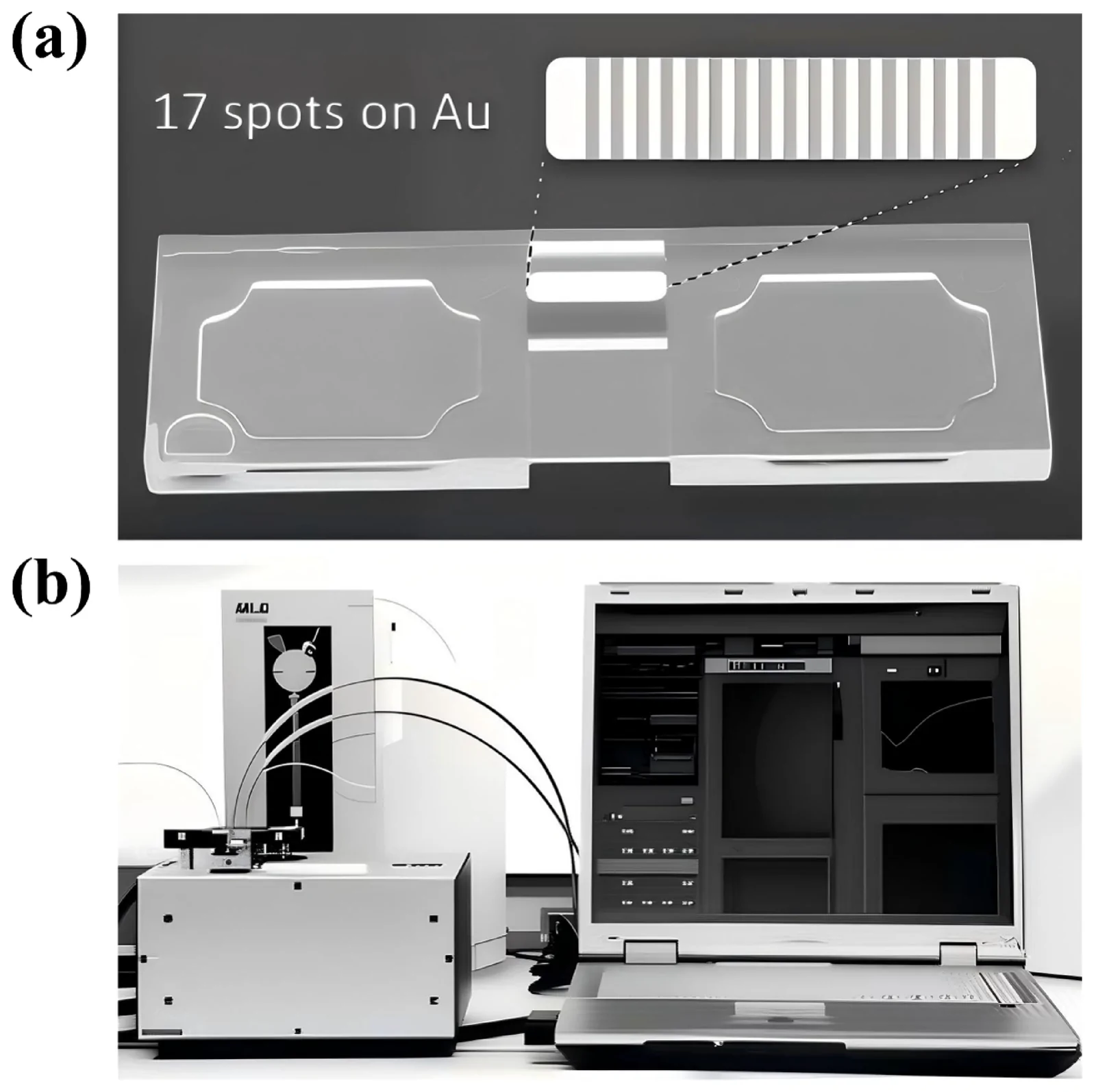
The Phytochip platform for on-chip detection of phytopathogenic RNA viruses. (**a**) A 17-spot gold sensor array integrated with microfluidic sample delivery. (**b**) Compact benchtop system combining SPR imaging, fluidic control, and real-time data acquisition [Experimentally demonstrated system, adapted from Ref. [64]].

**Figure 5:**
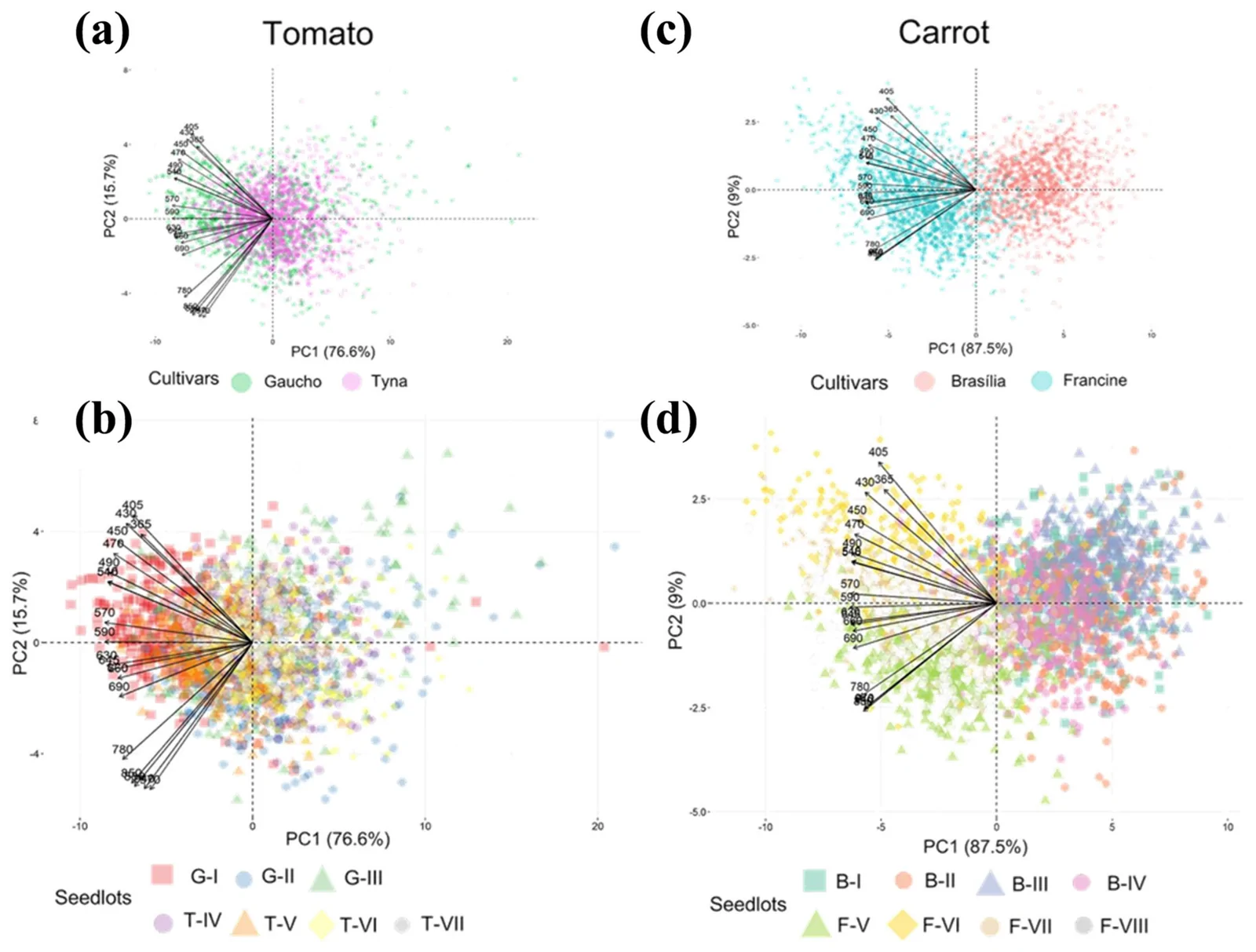
Principal component analysis (PCA) of multispectral seed imaging data for (**a**,**b**) tomato and (**c**,**d**) carrot, showing cluster discrimination of seed lots with differing vigor levels based on morphological and spectral features [Experimentally demonstrated system, adapted from Ref. [63]].

**Figure 6:**
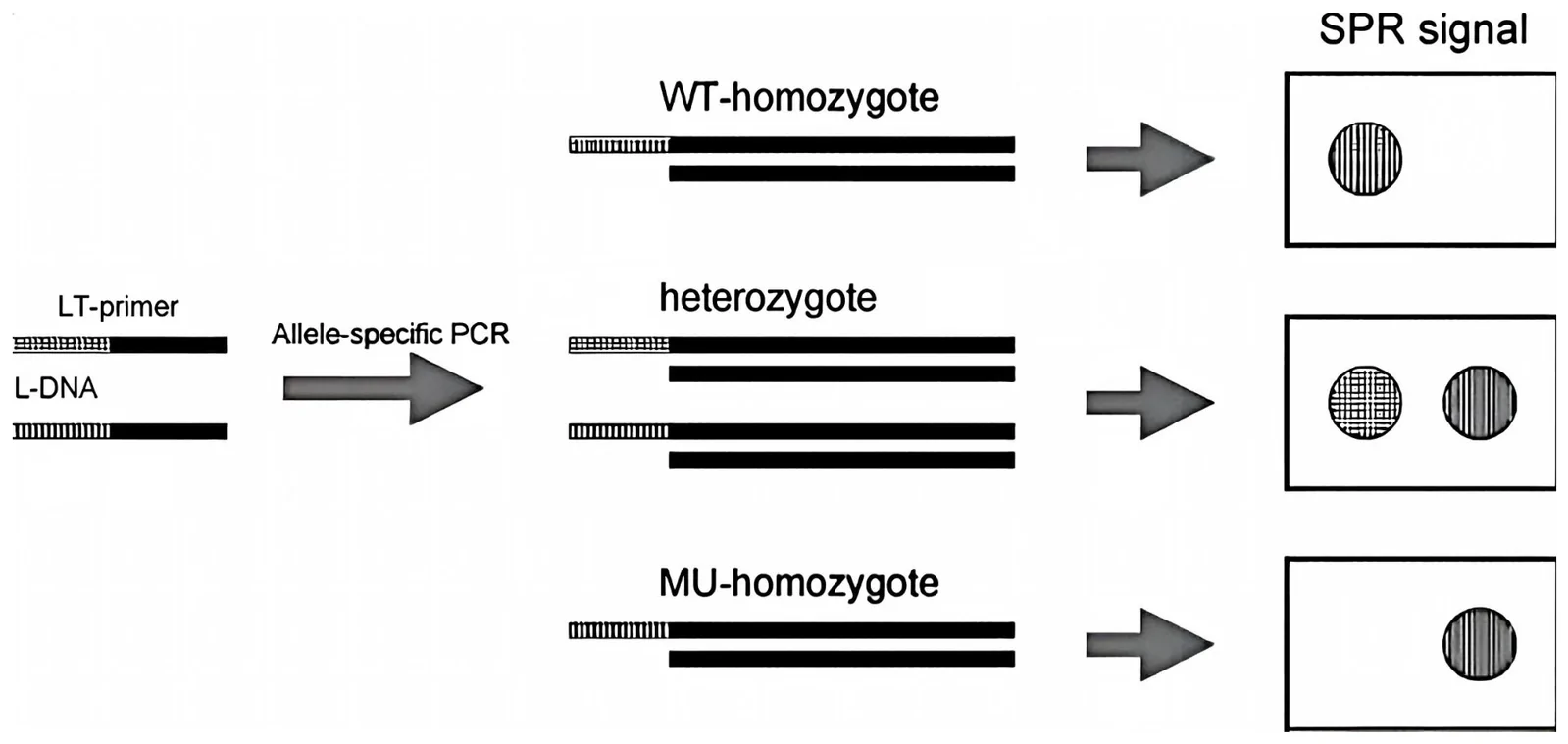
Panel integration paradigm for SPRi-based multidimensional seed vigor phenotyping. Spatially resolved multiplexed detection on an SPRi sensor surface. Multiple discrete detection spots are functionalized with distinct capture probes, and the imaging system records hybridization binding signals from each spot in parallel. This concept illustrates how SPRi can move beyond single-analyte detection toward multidimensional biomarker profiling [Conceptual architecture proposed in this work, extending Ref. [77]].

**Figure 7:**
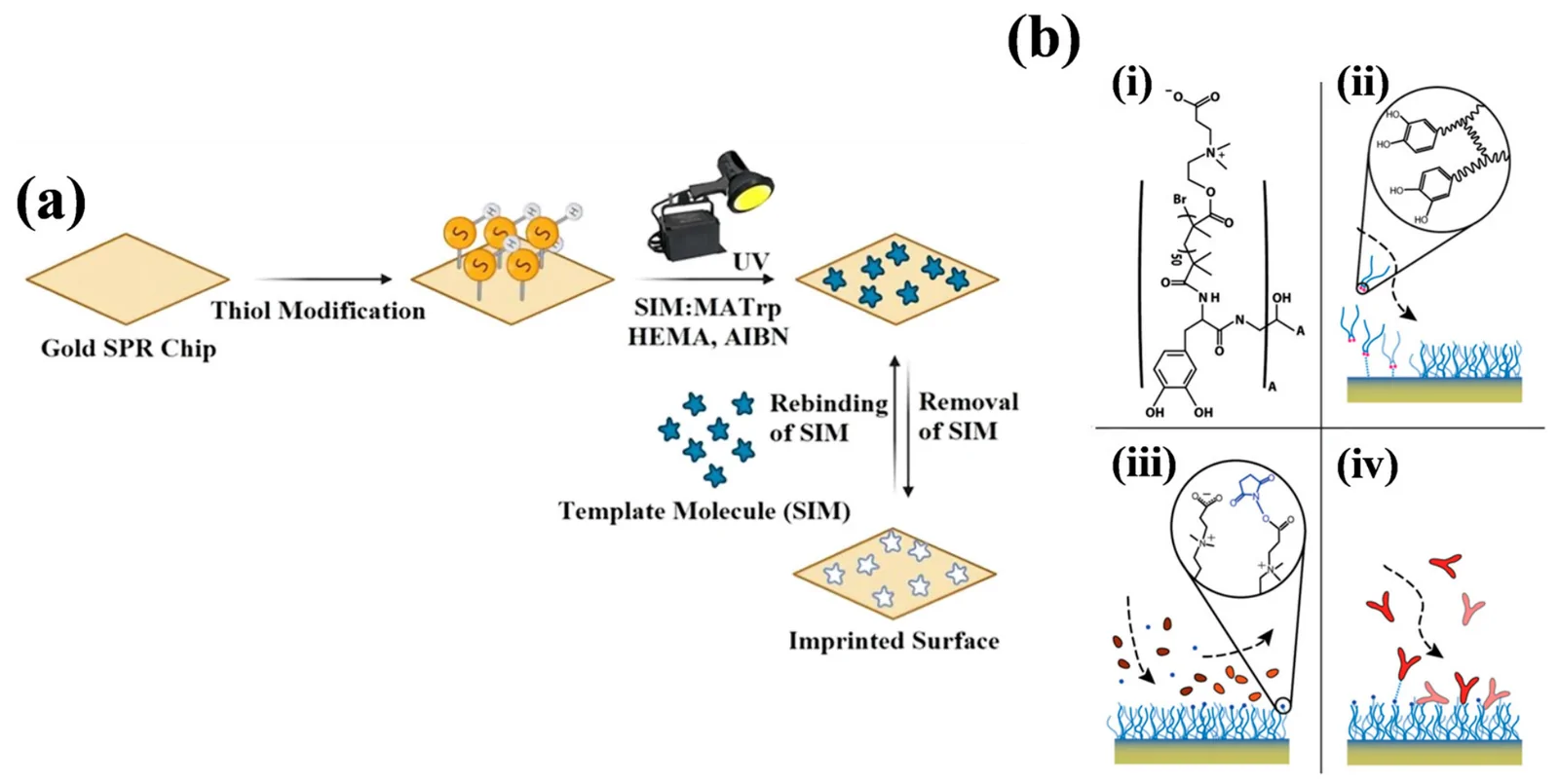
Surface functionalization strategies for complex seed matrices. (**a**) Stepwise fabrication of SIM-imprinted SPR sensor chips via in situ photopolymerization and subsequent template elution, providing synthetic recognition cavities for small-molecule markers [Experimentally demonstrated system, adapted from Ref. [82]]. (**b**) Four-step preparation of DOPA-PCBMA-modified surfaces for covalent antibody immobilization, demonstrating zwitterionic antifouling architecture [Experimentally demonstrated system, adapted from Ref. [95]].

**Figure 8:**
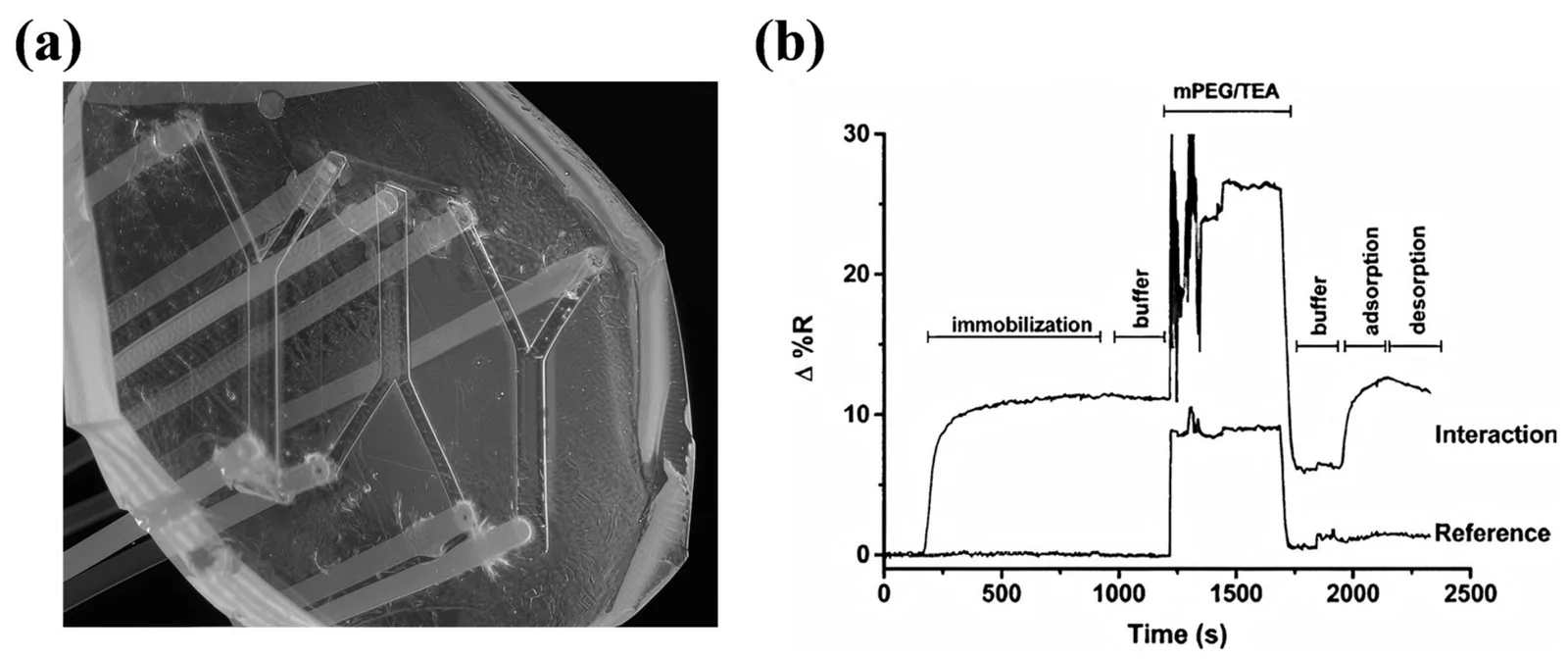
Microfluidic-enabled parallel kinetic fingerprinting on SPRi. (**a**) Photograph of a PDMS Y-shaped flow cell assembled on an SPRi gold sensor; the microfluidic network distributes sample to multiple detection regions for simultaneous analysis [Conceptual architecture proposed in this work, extending Ref. [104]]. (**b**) Real-time percent reflectivity changes recorded from signal (Interaction) and in-line reference (Reference) regions, showing immobilization, buffer equilibration, association, and desorption phases. The reference trace enables background subtraction essential for robust kinetic parameter extraction [Conceptual architecture proposed in this work, extending Ref. [104]].

**Figure 9:**
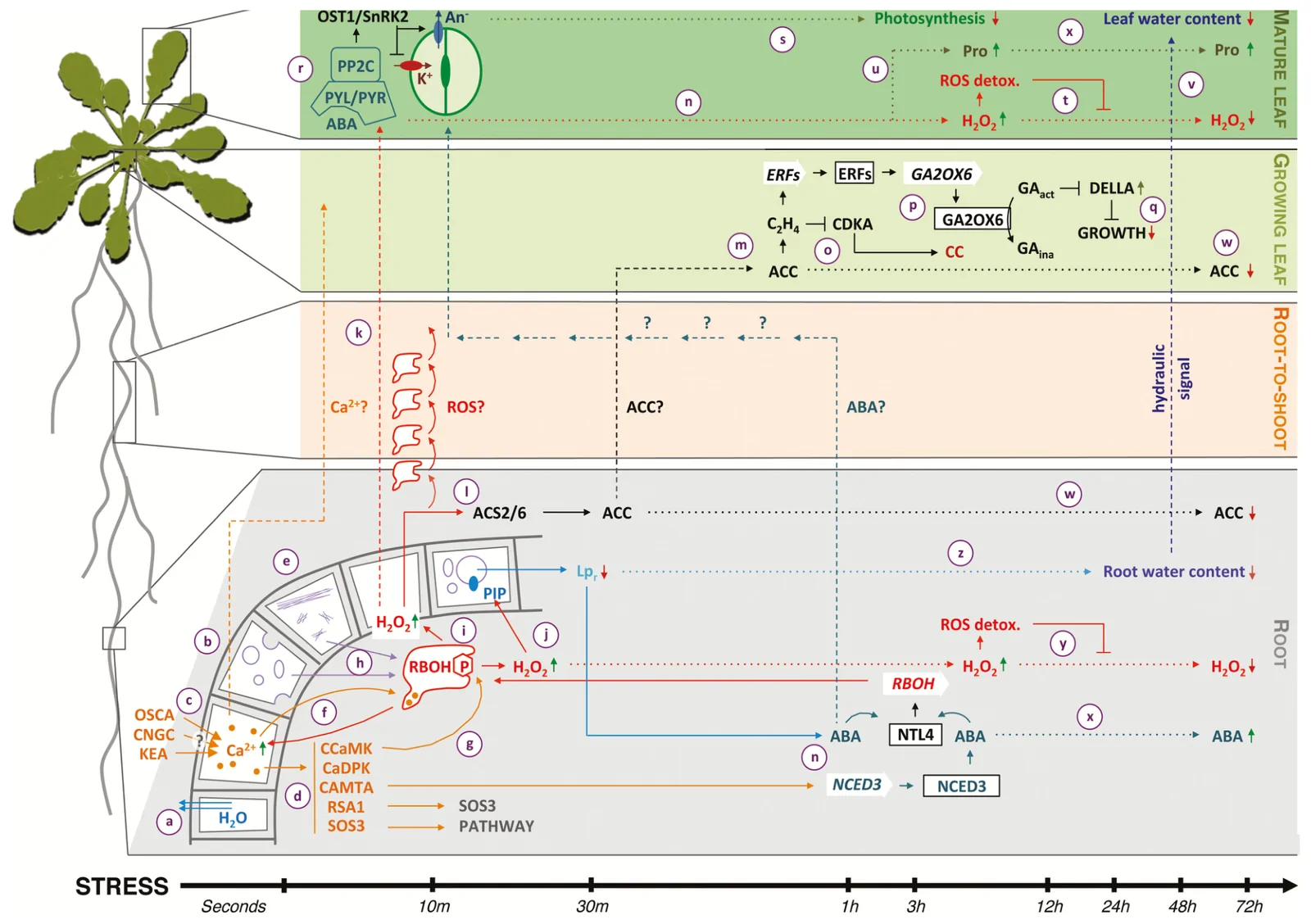
Sequential regulatory cascade of plant responses to osmotic stress, from cellular ionic and ROS signals to physiological phenotypes including growth arrest and photosynthetic decline [Experimentally demonstrated system, adapted from Ref. [105]].

**Figure 10:**
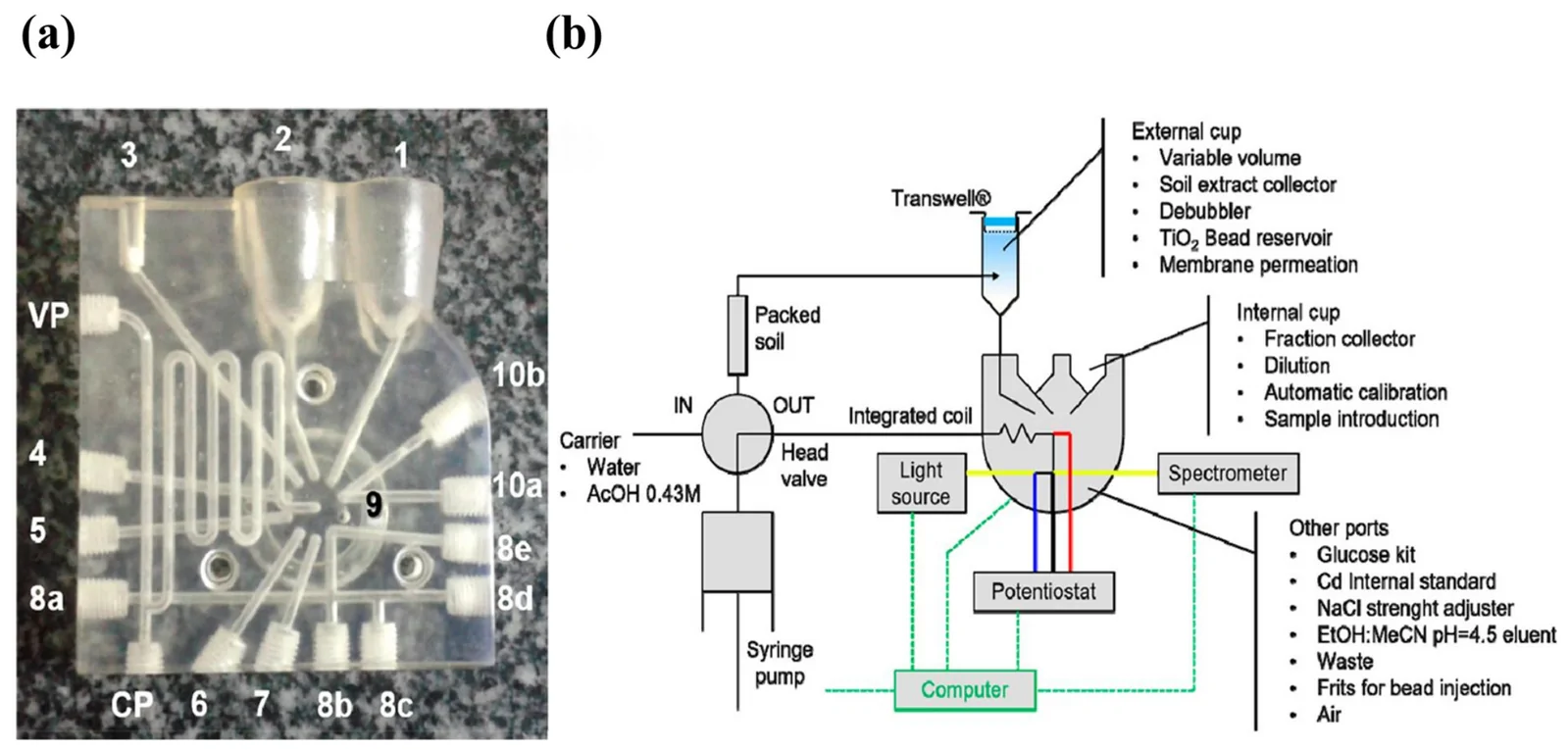
Microfluidic automation platforms for SPRi sample handling. (**a**) Close-up of a 3D-printed SLA-LOV manifold for in-valve biochemical assays and automated sample handling [Experimentally demonstrated system, adapted from Ref. [123]]. (**b**) Hardware layouts of 3D-printed LOV platforms supporting multiplexed detection configurations [Experimentally demonstrated system, adapted from Ref. [123]].

**Figure 11:**
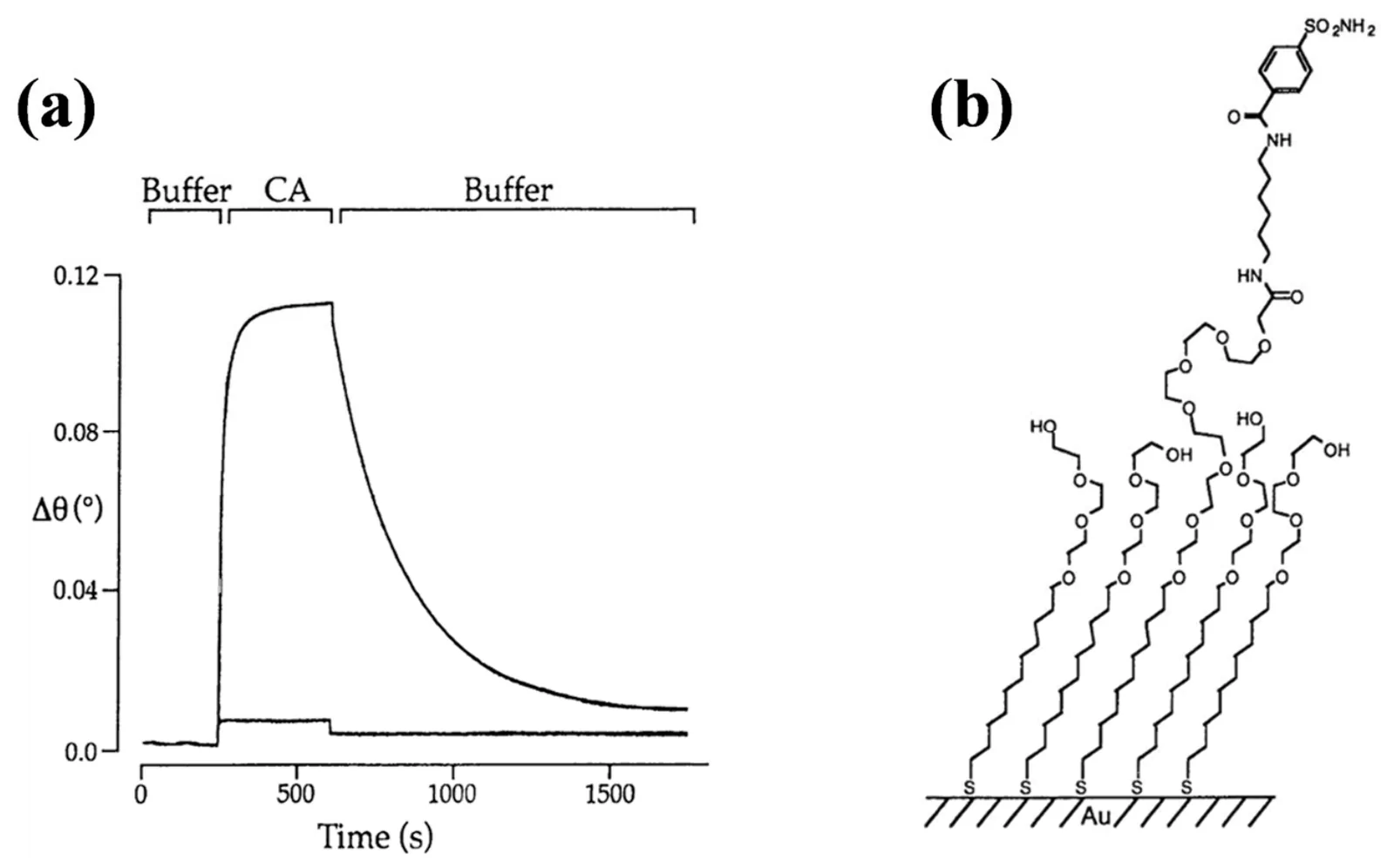
Self-assembled monolayer (SAM) surfaces for discriminating specific binding from non-specific background. (**a**) SPR sensorgrams recorded during sequential buffer–carbonic anhydrase (CA)–buffer injections: the pronounced response (upper trace) reflects specific binding of CA to the benzenesulfonamide-presenting SAM, whereas the flat trace on the non-functionalized monolayer shows only the trivial bulk refractive-index response [Experimentally demonstrated system, adapted from Ref. [129]]. (**b**) Schematic of the mixed SAM on gold presenting the benzenesulfonamide ligand within an oligo(ethylene glycol) matrix [Experimentally demonstrated system, adapted from Ref. [129]].

**Figure 12:**
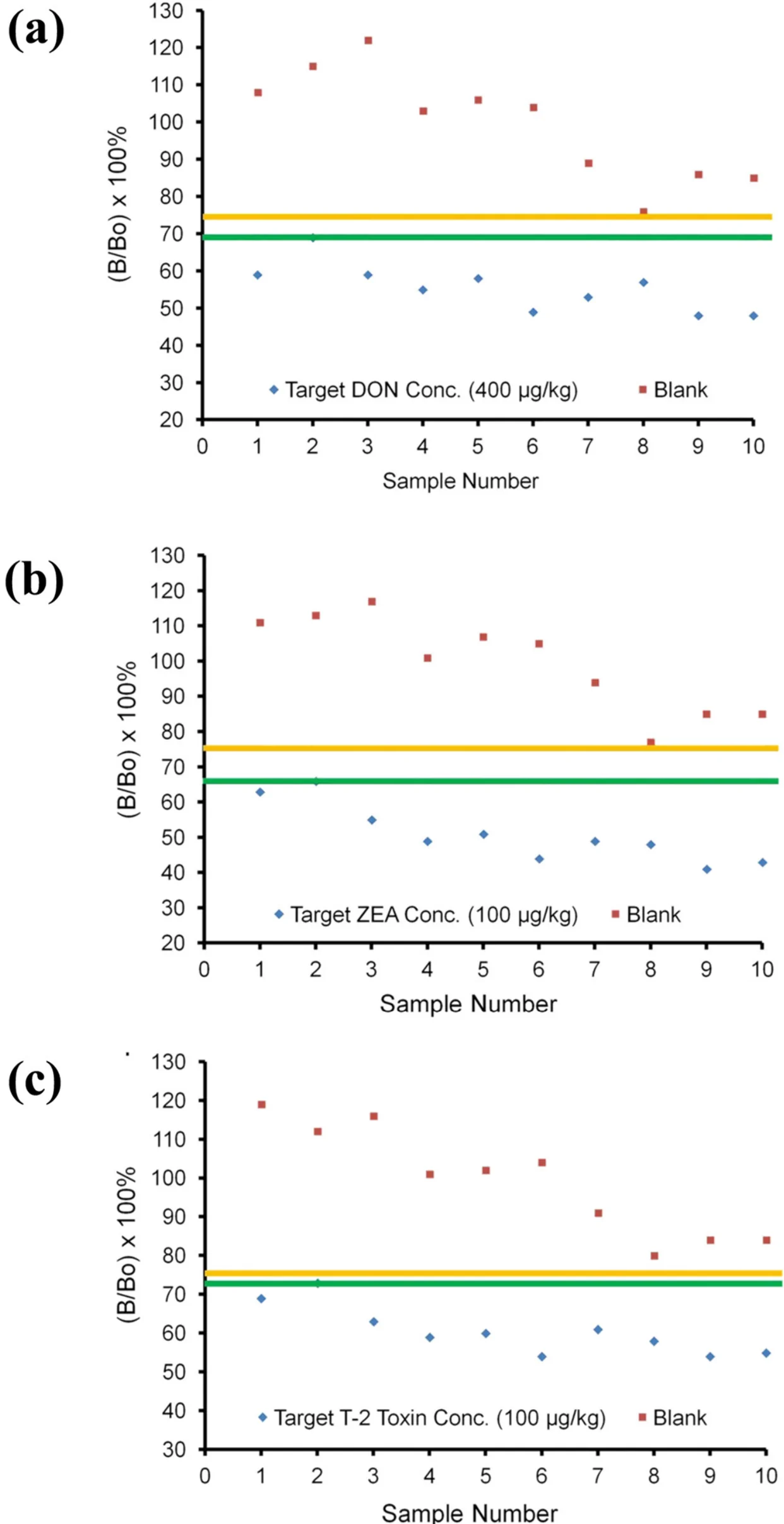
Verification of screening cut-off values for mycotoxins in wheat matrices using SPRi. Signal responses of blank (blue) and spiked (red) samples for (**a**) deoxynivalenol (DON), (**b**) zearalenone (ZEA), and (**c**) T-2 toxin, with horizontal lines indicating threshold levels [Experimentally demonstrated system, adapted from Ref. [134]].

**Figure 13:**
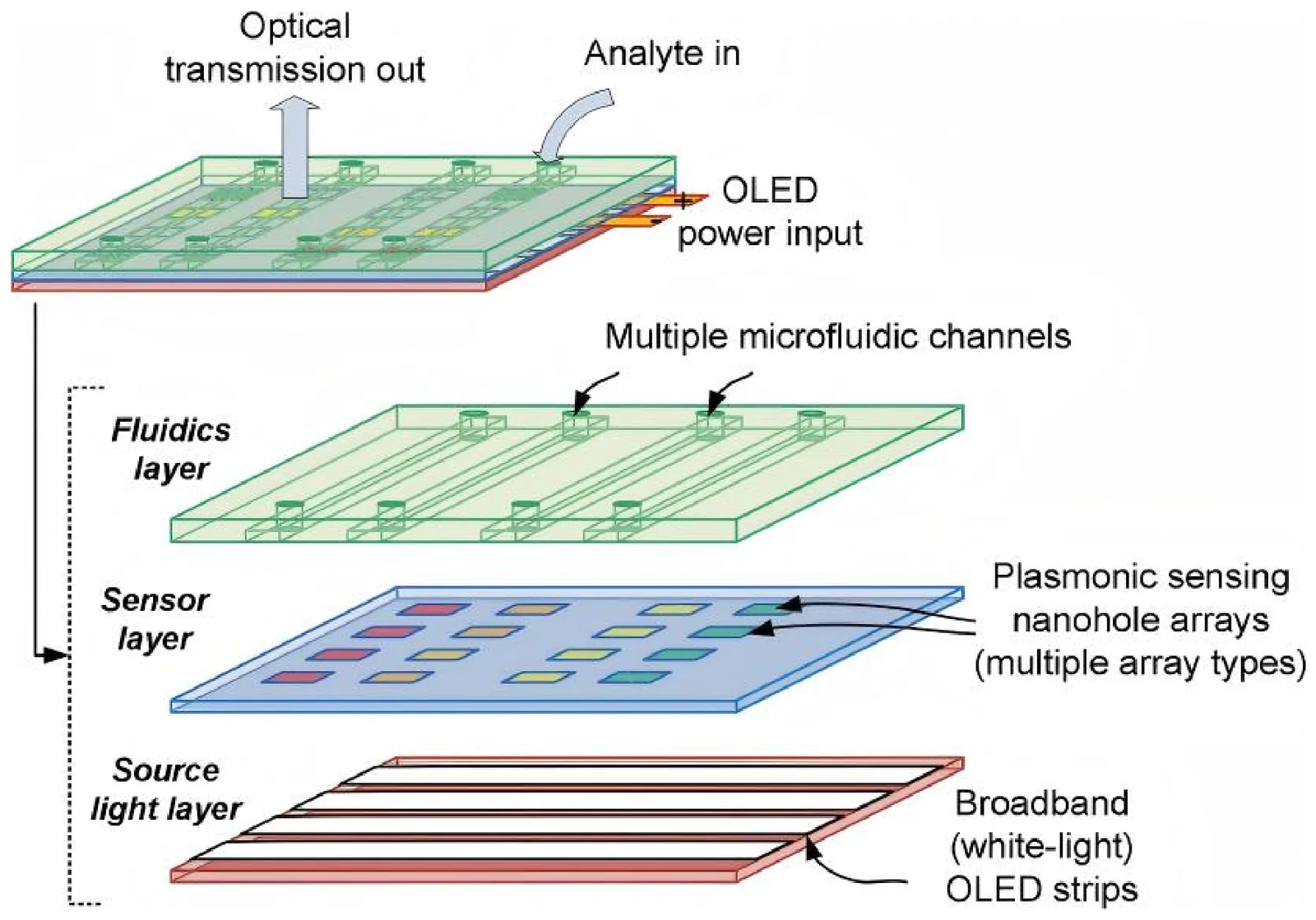
Three-layer stacked structure of a nanohole array integrated biochemical sensing platform. Exploded view showing the fluidic layer, plasmonic sensing layer with multiple nanohole arrays, and OLED source layer. Cross-sectional schematic of the integrated device [Experimentally demonstrated system, adapted from Ref. [137]].

**Figure 14:**
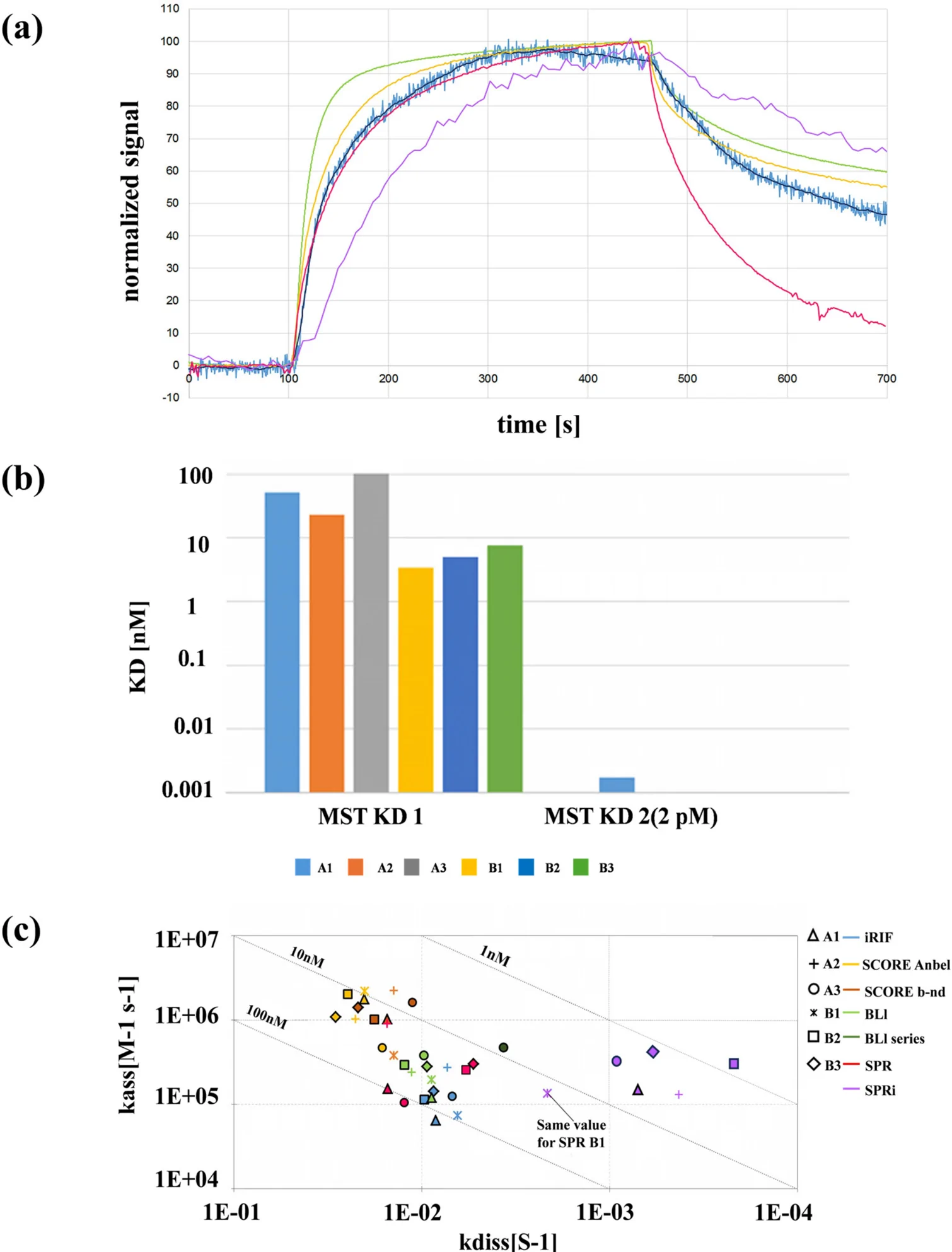
Cross-platform variability in label-free biosensing measurements. (**a**) Normalized binding curves of aptamer A3 recorded on five different label-free biosensing systems with varied time resolutions, showing divergent association and dissociation phase shapes [Experimentally demonstrated system, adapted from Ref. [138]]. (**b**) Equilibrium dissociation constants (KD) of the six thrombin aptamers (A1–B3) measured by microscale thermophoresis (MST) [Experimentally demonstrated system, adapted from Ref. [138]]. (**c**) Kinetic rate map (kass versus kdiss) of the same aptamer–target pair across iRIf, SCORE, BLI, SPR, and SPRi platforms, with diagonal lines indicating constant KD, revealing systematic ranking differences between platforms [Experimentally demonstrated system, adapted from Ref. [138]].

**Table 6:** Principal interferent classes in seed-derived matrices, interference mechanisms, and mitigation strategies.

Interferent	Interference Mechanism	Mitigation Strategy	Status in Seed Matrices
Non-specific proteins	Adsorption on gold; false binding signal	Zwitterionic brushes; OEG SAMs [75,95,129,130]	Demonstrated in serum/food; seed exudates to verify
Polysaccharides	Viscosity; channel clogging; nanoparticle trapping	On-chip microfiltration; controlled dilution	Partially addressed; fouling duty cycles unquantified
Polyphenols/phenolics	Strong surface adsorption; coating degradation	Poly(carboxybetaine) layers; accelerated fouling tests	Long-term stability unquantified
Salts/ionic strength	Bulk refractive-index shift mimics binding	In-line reference spots; matched buffers [111]	Reference-uniformity assumption to verify
Particulate debris	Clogging; heterogeneous fouling	0.22 μm upstream filtration; centrifugal cartridges	Engineering mitigation demonstrated; seed-specific validation pending

## Data Availability

No new data were created or analyzed in this study. Data sharing does not apply to this article.
